# Degradation of the ABA co-receptor ABI1 by PUB12/13 U-box E3 ligases

**DOI:** 10.1038/ncomms9630

**Published:** 2015-10-20

**Authors:** Lingyao Kong, Jinkui Cheng, Yujuan Zhu, Yanglin Ding, Jingjing Meng, Zhizhong Chen, Qi Xie, Yan Guo, Jigang Li, Shuhua Yang, Zhizhong Gong

**Affiliations:** 1 State Key Laboratory of Plant Physiology and Biochemistry, College of Biological Sciences, China Agricultural University, Beijing 100193, China; 2 State Key Laboratory of Plant Genomics, Institute of Genetics and Developmental Biology, Chinese Academy of Sciences, Beijing 100101, China; 3 National Center for Plant Gene Research, Beijing, 100193, China

## Abstract

Clade A protein phosphatase 2Cs (PP2Cs) are abscisic acid (ABA) co-receptors that block ABA signalling by inhibiting the downstream protein kinases. ABA signalling is activated after PP2Cs are inhibited by ABA-bound PYR/PYL/RCAR ABA receptors (PYLs) in *Arabidopsis*. However, whether these PP2Cs are regulated by other factors remains unknown. Here, we report that ABI1 (ABA-INSENSITIVE 1) can interact with the U-box E3 ligases PUB12 and PUB13, but is ubiquitinated only when it interacts with ABA receptors in an *in vitro* assay. A mutant form of ABI1-1 that is unable to interact with PYLs is more stable than the wild-type protein. Both ABI1 degradation and all tested ABA responses are reduced in *pub12 pub13* mutants compared with the wild type. Introducing the *abi1-3* loss-of-function mutation into *pub12 pub13* mutant recovers the ABA-insensitive phenotypes of the *pub12 pub13* mutant. We thus uncover an important regulatory mechanism for regulating ABI1 levels by PUB12 and PUB13.

## Abstract (web summary)

Signaling by the plant hormone abscisic acid (ABA) is regulated by the ABI1 protein phosphatase. Here Kong *et al.* propose that ABA signaling is fine-tuned by ubiquitination of ABI1 which promotes ABI degradation in response to ABA.

## Graphical abstract

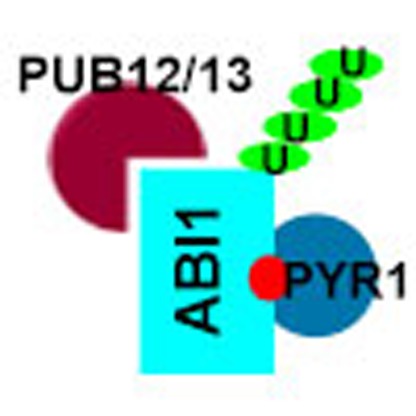

Abscisic acid (ABA) is a plant hormone that regulates seed dormancy, seed germination, seedling growth, as well as biotic and abiotic stress responses^1^,^2^. Like other plant hormone signalling pathways^3^, the ABA signalling pathway follows a ‘relief of repression' model for signal transduction. The clade A protein phosphatase 2Cs (PP2Cs) play a central role in negatively regulating ABA signalling^4^,^5^. The cytoplasmic PYR (Pyrabactin Resistance)/PYL (Pyrabactin Resistance 1-Like)/RCAR (Regularly Component of ABA Receptors) ABA receptors (PYLs) bind to ABA and interact with PP2Cs^6^,^7^, thereby releasing PP2C inhibition of ABA-activated protein kinases OST1 (SnRK2.6)/SnRK2.2/2.3 (refs 7, 8, 9), GHR1 (ref. 10) and SnRK1 (ref. 11), and also some calcium-dependent protein kinases^12^,^13^,^14^. These protein kinases phosphorylate and activate downstream targets such as ABF (ABRE BINDING FACTOR) transcriptional factors to control gene expression in the nucleus; they also phosphorylate and activate the key anion channel SLAC1 in guard cells to control stomatal movement^9^,^10^,^12^,^13^. The ABA-binding affinities of PYLs are enhanced when they interact with PP2Cs, so that PP2Cs are also considered as ABA co-receptors in ABA signalling^15^,^16^. Some PYLs can also interact with PP2Cs in an ABA-independent manner, but their inhibition of PP2Cs is weaker than that of PYLs binding to ABA^17^. Although research has established that these PP2Cs are regulated by ABA receptors, whether they are modulated by other factors is largely unknown^18^.

In this study, we demonstrate that ABI1 (ABA-INSENSITIVE 1), a key PP2C protein in ABA signalling in *Arabidopsis*, is degraded by the 26S proteasome pathway. Two U-Box E3 ligases, PUB12 (AT2G28830) and PUB13 (AT3G46510), interact with ABI1 but ubiquitinate ABI1 only when ABI1 interacts with PYLs in an *in vitro* assay. This study uncovers a novel regulatory mechanism that dynamically modulates the key negative regulator ABI1 in the ABA signalling pathway.

## Results

### ABI1 is degraded by 26S proteasomes

**Figure 1:**
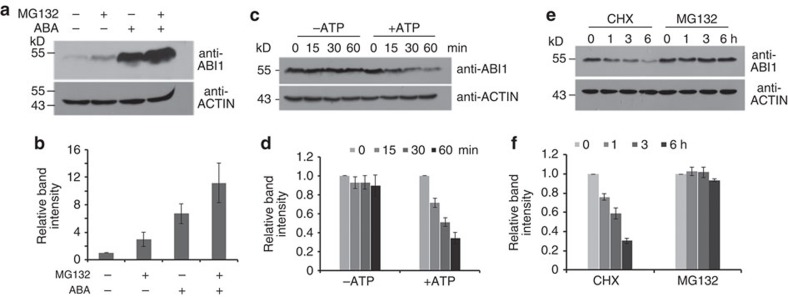
ABI1 degradation is mediated by the 26S proteasome pathway. (**a**) Treatment with the 26S proteasome inhibitor MG132 greatly increases the level of ABI1. Wild-type seedlings were treated with 50 μM MG132 or H_2_O for 6 h, or 50 μM ABA plus 50 μM MG132 or H_2_O for 6 h, and then total proteins were extracted and used for immunoblotting analysis with anti-ABI1 antibody. ACTIN protein was used as a loading control. (**b**) Quantitative analysis of the band intensity in **a**. The abundance of ABI1 at the start (ABA-, MG132-) was set to 1 as a reference for calculating relative abundance of various treatment. Error bars means±s.e.m. (*n*=3 independent experiments). (**c**) ABI1 degradation is enhanced by addition of ATP. Wild-type seedlings were treated with 50 μM ABA for 6 h, then total proteins were isolated and incubated with or without 1 mM ATP for different times, and subjected to immunoblotting analysis with anti-ABI1 antibody. ACTIN protein was used as a loading control. (**d**) Quantitative analysis of the band intensity in **c**. The abundance of ABI1 at the 0 min (ATP−, ATP+) was set to 1, respectively. The values were references for calculating relative abundance after various treatment time. Error bars are means±s.e.m. (*n*=3 independent experiments). (**e**) Addition of the protein biosynthesis inhibitor cycloheximide (CHX) does not change the degradation pattern of ABI1. Wild-type seedlings were treated with 50 μM ABA for 6 h firstly. After washing away excess of ABA, the seedlings were treated with 100 μM CHX or 50 μM MG132 separately for different times before protein was isolated for western blot with anti-ABI1 antibody. ACTIN was used as a loading control. (**f**) Quantitative analysis of the band intensity in **e**. The abundance of ABI1 at the 0 h (CHX, MG132) was set to 1, respectively. The values were references for calculating relative abundance after various treatment time. Error bars are means±s.e.m. (*n*=3 independent experiments).

Proteolysis is critical for regulating the turnover of key regulatory proteins in plants^19^. To determine whether ABI1 is regulated by 26S proteasomes, we used immunoblotting to measure the ABI1 level after seedlings were treated with MG132 (an inhibitor of 26S proteasomes). Immunoblotting analysis with anti-ABI1 antibody (see Supplementary Fig. 1 for ABI1 antibody specificity) indicated that ABI1 accumulation was higher in seedlings treated with MG132 than the control (without MG132; Fig. 1a,b). ABA treatment significantly increased ABI1 level comparing without ABA treatment. As ABI1 protein level is very low under normal growth condition, in the next experiments we used the proteins isolated from ABA-treated seedlings. Because ATP can enhance the protein degradation rate in a cell-free 26S proteasome assay, addition of ATP to total proteins enhanced the degradation rate of ABI1 (Fig. 1c,d). To exclude the translational effect, we treated seedlings with a protein biosynthesis inhibitor cycloheximide (CHX, 100 μM) to block the protein biosynthesis, so that the only changes would be already translated proteins. The results indicated that ABI1 was degraded more quickly with CHX treatment than with MG132 (Fig. 1e,f). These results suggest that the turnover of ABI1 protein is mediated by 26S proteasome pathway.

### The U-box E3 ligases PUB12 and PUB13 can interact with ABI1

**Figure 2:**
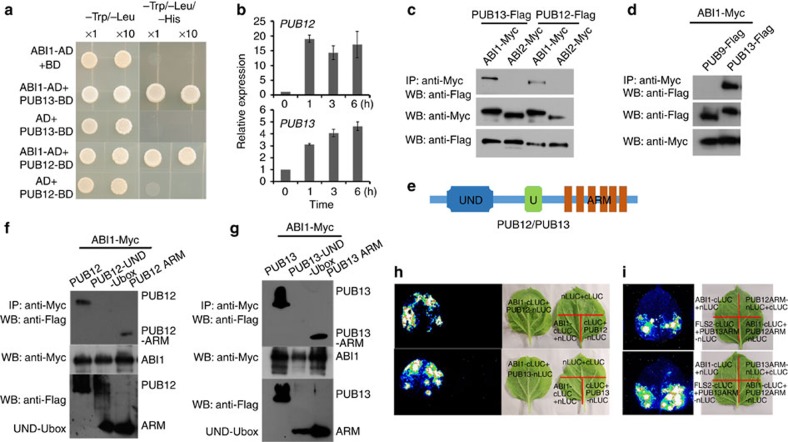
ABI1 can interact with PUB12 and PUB13. (**a**) ABI1 interacts with PUB12 and PUB13 in a yeast two-hybrid assay. AD: Gal4 activation domain; BD: Gal4 DNA-binding domain. 2D: synthetic dropout medium without Trp and Leu; 3D: synthetic dropout interaction medium without Trp, Leu and His. (**b**) The expression of *PUB12* and *PUB13* is induced by ABA treatment. RNAs were isolated from 7-day-old seedlings treated with 50 μM ABA for different times. Three independent experiments were done, each with three replicates. (**c**) ABI1, but not ABI2, interacts with PUB12 and PUB13 in a Co-IP assay. ABI1-Myc and PUB12- or PUB13-Flag plasmids, or ABI2-Myc and PUB12- or PUB13-Flag plasmids, were co-transfected into *Arabidopsis* protoplasts. Co-IP was carried out with anti-Myc agarose from total isolated proteins, and immunoblotting analysis was done with anti-Flag and anti-Myc antibody. (**d**) ABI1 does not co-immunoprecipitate PUB9 but co-immunoprecipitates PUB13 in the *Arabidopsis* protoplast transient expression assay. ABI1-Myc and PUB9-Flag or PUB13-Flag plasmids were co-expressed in protoplasts. Co-IP and immunoblotting analysis were done as in **c**. (**e**) The domain composition of PUB12/13 proteins. UND, U-box N-terminal domain; U, U-box domain; ARM, Armadillo repeat domain. (**f**) ABI1 co-immunoprecipitates the ARM domain of PUB12 but not UND-U-box in the *Arabidopsis* protoplast transient expression assay. ABI1-Myc and ARM-Flag, ABI1-Myc and UND-U-box-Flag, or ABI1-Myc and PUB12-Flag plasmids were co-expressed in protoplasts. Co-IP and immunoblotting analysis were done as in **c**. (**g**) ABI1 interacts with the ARM domain of PUB13. The assay was similar to that in **f**. (**h**) ABI1 can interact with PUB12 or PUB13 as indicated by the split firefly luciferase complementation imaging (LCI) assay. ABI1 translationally fused with the C terminus of LUC (ABI1-cLUC) was co-expressed with PUB13 translationally fused with the N-terminus of LUC (PUB13-nLUC) by co-infiltrating *Agrobacterium* carrying different plasmids into *Nicotiana benthamiana* leaves. Images were collected 3 d after *Agrobacterium* infiltration. (**i**) ABI1 can interact with the ARM domain of PUB12 or PUB13 as indicated by the LCI assay as in **h**. The interaction of FLS2-cLUC with PUB13 ARM-nLUC was used as a positive control.

To determine which E3 ubiquitin ligases target ABI1, we performed yeast two-hybrid assays. We selected the following candidates, which have been shown to be involved in ABA signalling: DWA1 (DWD (CULLIN 4-DAMAGED DNA BINDING 1-DDB1 BINDING WD40) HYPERSENSITIVE TO ABA1), DWA2, RGLG1/2 (THE MEMBRANE-ASSOCIATED RING DOMIAN LIGASE1/2), SDIR1 (SALT- AND DROUGHT-INDUCED RING FINGER1) and KEG (KEEP ON GOING)^20^,^21^,^22^,^23^,^24^,^25^,^26^,^27^. We also selected some plant U-box E3 ligases (PUBs)^28^. The *Arabidopsis* genome contains 64 genes encoding PUBs, the functions of which are mostly unknown^28^. In total, we tested 29 proteins (including 23 PUB proteins) and found that five proteins (PUB12, PUB13, PUB44, PUB60 and SDIR1) interacted with ABI1 in the yeast two-hybrid assay (Supplementary Fig. 2). Finally, we selected PUB12 and PUB13 for further characterization because these two proteins interacted with ABI1 in both the yeast two-hybrid assay (Fig. 2a) and in other assays, as described later.

PUB12 and PUB13, two highly homologous U-box E3 ligases, are involved in the regulation of FLS2 turnover^29^, and PUB13 is also involved in defence response, cell death and flowering^30^. The expression of *PUB12* and *PUB13* was induced by ABA treatment (Fig. 2b). Histochemical β-glucuronidase (GUS) activity assays indicated that GUS was widely expressed in all tissues including leaves, roots and guard cells in transgenic plants carrying either *PUB12* or *PUB13* promoter driving *GUS* (Supplementary Fig. 3). An co-immunoprecipitation (Co-IP) assay using proteins extracted from *Arabidopsis* protoplasts transiently transfected with different plasmids indicated that PUB12-Flag or PUB13-Flag co-immunoprecipitated ABI1-Myc but not ABI2-Myc (Fig. 2c) or other ABI1 homologues, including HAB1, HAB2, AHG1 and AHG3 (refs 31, 32, 33; Supplementary Fig. 4). As a negative control, PUB9-Flag did not co-immunoprecipitate ABI1-Myc (Fig. 2d). To determine the possibility that ABI1 interacts with PUB12/13 *in vivo*, we carried out liquid chromatography–tandem mass spectrometry (LC–MS/MS) analysis using affinity purified proteins from *ProABI1:ABI1-Flag* seedlings with anti-Flag antibody. Peptides corresponding to PUB12 were identified in this assay (Supplementary Data 1). We also found that native ABI1 could co-immunoprecipitate PUB13-Flag from transgenic plants expressing *Pro35S:PUB13-Flag* (Supplementary Fig. 5). On the basis of these results we suggest that ABI1 is capable of forming a complex with PUB12 and/or PUB13 *in vivo*. Protein deletion analysis indicated that ABI1 could interact with the Armadillo repeat domain (ARM) domain but not with UND-U-box (U-box N-terminal domain) of PUB12/13 (Fig. 2e–g). A firefly luciferase complementation imaging assay based on transient expression^34^ suggested ABI1 may interact with either the whole PUB12 or PUB13 protein (Fig. 2h) and with the ARM domain (Fig. 2i). The interaction of FLS2 with the PUB13 ARM domain was used as a positive control^29^. These results indicate that ABI1 can specifically interact with the ARM domain of PUB12/13.

### PUB12/13 mediate ABI1 ubiquitination *in vitro*

**Figure 3:**
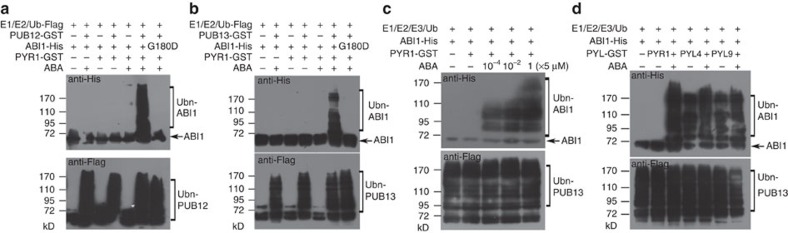
**PUB12 and PUB13 ubiquitinate ABI1 in an** ***in vitro*** **assay.** (**a**,**b**) PUB12 and PUB13 ubiquitinate ABI1 depending on the addition of both PYR1 and ABA in the assays. Different proteins purified from *E. coli* were added to the ubiquitination reaction buffer with 5 μM ABA or without ABA. The hypermorphic mutation ABI1-1 (ABI1^G180-D^) protein was included in the reaction. ABI1 ubiquitination was detected with anti-His antibody, and the overall ubiquitination was detected with anti-Flag antibody. (**c**) Increasing ABA concentration increases ubiquitinated ABI1 level. The same amount of different proteins (here–PUB13was used as an E3 ligase) were included in the ubiquitination reactions with addition of 0, 5 × 10^−4^, 5 × 10^−2^ or 5 μM ABA, respectively. ABI1 ubiquitination was detected with anti-His antibody, and the overall ubiquitination was detected with anti-Flag antibody. (**d**) PUB13 ubiquitinates ABI1 in presence of PYL4 and PYL9 with or without addition of ABA. Different proteins purified from *E. coli* were added to ubiquitination buffer with 5 μM ABA or without ABA. PYL4 and PYL9 interact with ABI1 in the absence of ABA, while PYR1 interacts with ABI1 only in the presence of ABA. ABI1 ubiquitination was detected by anti-His antibody, and the activity of PUB13 E3 ligase was detected by anti-Flag antibody. Whether or not ABA was added, ABI1 was ubiquitinated with addition of PYL4 or PYL9. However, addition of ABA slightly increases ubiquitination level compared with no addition of ABA.

We then used an *in vitro* ubiquitination assay^29^ to test whether PUB12 or PUB13 could ubiquitinate ABI1. All proteins including E1, E2, GST (glutathione S-transferase)-tagged PUB12 (PUB12-GST) or PUB13-GST, ABI1-His and PYR1-GST protein were purified from *Escherichia coli*, and Flag-tagged ubiquitin (Ub-Flag) is a commercial product. The *abi1-1* mutation is hypermorphic, and *abi1-1* mutant shows pleiotropic ABA-insensitive phenotypes in all tested ABA responses^4^,^5^. The mutation of G180 to D180 in *abi1-1* blocks the interaction of ABI1^G180-D^ (ABI1-1) with PYL ABA receptors^6^. The mutated protein ABI1-1-His purified from *E. coli* was also included in the assays. Consistent with previous results^29^, both PUB12 and PUB13 possessed auto-ubiquitination activity when recombinant E1, E2, Ub-Flag and ATP were added (Fig. 3a,b). Although ABI1-His was added to these two reactions combining with either addition of PYR1 or 5 μM ABA, the ladder-like ubiquitinated ABI1-His could not be detected. Only when both PYR1 and ABA were added together in the ubiquitination reaction, the ladder-like arrangement of proteins with anti-His antibody could be detected, indicating that both PUB12 and PUB13 ubiquitinated ABI1-His (Fig. 3a,b). In contrast, ABI1-1-His was not ubiquitinated in these assays (Fig. 3a,b). We observed that when ABA concentration was increased from 5 × 10^−4^ to 5 μM, the ubiquitination strength of ABI1-His was gradually increased (Fig. 3c), suggesting that ABI1 ubiquitination relies on ABA concentration in presence of PYR1.

PYL ABA receptors can be divided into two subgroups according to their interaction with PP2Cs. One group includes PYR1 and PYL1-3 that interact with and inhibit PP2Cs only after they bind to ABA. The other group includes PYL4-10 that can interact with and inhibit PP2Cs without binding to ABA, but their inhibition of PP2Cs is stronger after they bind to ABA^17^. We selected PYL4 and PYL9 from the latter group to determine whether ABI1 can be ubiquitinated by PUB13 when either PYL4 or PYL9 is available in the *in vitro* ubiquitination assay using proteins purified from *E. coli* as performed above. Immunoblotting analysis with anti-His antibody revealed that ABI1-His could be ubiquitinated with or without ABA (5 μM) in the presence of PYL4-GST or PYL9-GST in the ubiquitination assays (Fig. 3d). However, the ubiquitination levels were slightly higher with addition of ABA than without ABA. PYR1 with or without addition of ABA (5 μM) was used as controls. These results suggest that PUB13-mediated ABI1 ubiquitination depends on the interaction of ABI1 with ABA receptors in the *in vitro* assay. PUB13-mediated ABI1 ubiquitination in presence of PYL4 and PYL9 without ABA suggests that ABI1 may be also dynamically regulate at protein level even under normal conditions.

### PUB12/13 are required for ABI1 degradation

**Figure 4:**
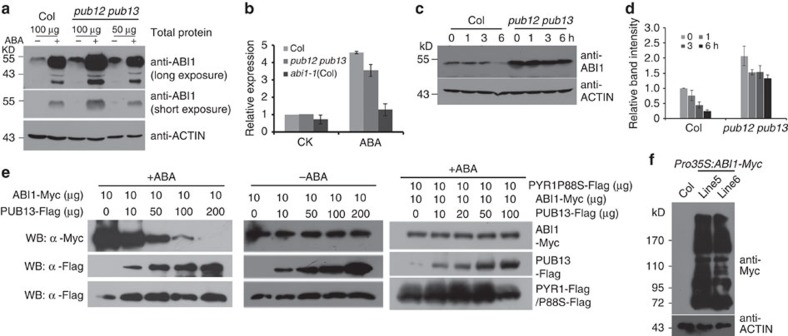
PUB12/13 are required for ABI1 degradation in plant cells. (**a**) ABI1 level is higher in the *pub12 pub13* double mutant than in the wild type. The total proteins extracted from wild-type plants or the *pub12 pub13* mutant treated with or without 50 μM ABA for 6 h were used for immunoblotting analysis with anti-ABI1 antibody. Total proteins from the *pub12 pub13* mutant were diluted from 100 to 50 μg, and short and long exposure time were used in order for comparison. ACTIN was used as a loading control. (**b**) Relative expression of *ABI1* in the wild type (Col), the *pub12 pub13* mutant and *abi1-1* (Col). Total RNAs extracted from 7-day-old seedlings treated with 50 μM ABA for 1 h were used for real-time RT–PCR. (**c**) Comparison of degradation between the *pub12 pub13* mutant and the wild type. The 7-day-old seedlings were treated with 100 μM CHX for different times. At each time point, total proteins were extracted and used for immunoblotting analysis with anti-ABI1 antibody. ACTIN was used as a loading control. (**d**) Quantitative analysis of the signal intensity in **c**. The abundance of ABI1 at the 0 h was set to 1 as a reference for calculating relative abundance of various time point. Error bars are means±s.e.m. (*n*=3 independent experiments). (**e**) PUB13-mediated ABI1 degradation requires ABA as well as ABI1 interaction with PYR1. The protoplasts from a transgenic line overexpressing *PYR1-Flag* or the wild type seedling were co-expressed with 10 μg of *pro35S:ABI1-Myc* and different amounts of *pro35S:PUB13-Flag* plasmids (0 to 200 μg), or together with 10 μg of *Pro35S-PYR1P88S* (only for wild type protoplasts) for 16 h and treated with 10 μM ABA (left, right) or without ABA (middle) for 4 h before immunoblotting analysis was performed using anti-Myc antibody or anti-Flag antibody. PYR1-Flag was used as the loading control. (**f**) ABI1 is ubiquitinated in plants. Ubiquitinated proteins were enriched from P62-agarose matrix that was incubated with total proteins isolated from two independent transgenic plants stably expressing ABI1-Myc or from wild-type plants. Plant materials were pretreated with 50 μM ABA for 12 h and 50 μM MG132 for 6 h before immunoblotting analysis was performed using anti-Myc antibody.

To determine whether PUB12 and PUB13 modulate ABI1 degradation in plant cells, we compared ABI1 protein level between *pub12 pub13* mutant and the wild type using anti-ABI1 antibody. A previous study indicated that the transcription of *PUB12* in *pub12* (*pub12-2* mutant) is significantly reduced and *pub13* is a null mutant allele^29^. Immunoblotting analysis indicated that more ABI1 accumulated in the *pub12 pub13* mutant than in the wild type with or without ABA treatment (Fig. 4a). As *ABI1* transcripts were lower in the *pub12 pub13* mutant than in the wild type, but higher than *abi1-1* (Col) (the same mutation as the *abi1-1* in Ler)^35^ (Fig. 4b, see also RNA-seq data in Supplementary Data 2 and 3), the higher accumulation of ABI1 protein in the *pub12 pub13* mutant than the wild type may be attributed to post-transcriptional regulation. In order to examine the effect of PUB12/13 on ABI1 protein degradation in plants, we treated seedlings with 100 μM CHX to block protein translation and then performed an immunoblotting assay with anti-ABI1 antibody. As shown in Fig. 4c,d, the degradation of ABI1 protein occurred more slowly in the *pub12 pub13* mutant than wild type.

To test the effect of increasing PUB13 on ABI1 stability in plant cells, we transiently co-transfected transgenic *Pro35S:PYR1-Flag Arabidopsis* protoplasts with *Pro35S:ABI1-Myc* plus increasing amount of *Pro35S:PUB13-Flag* plasmids (Fig. 4e). Here *Pro35S:PYR1-Flag* transgenic plants were used as we consider that more PYR1 proteins are required when more ABI1 proteins are expressed in this assay. After the protoplasts were cultured for 16 h, and then treated with or without 10 μM ABA for 4 h, the total proteins were extracted and used for immunoblotting analysis using anti-Myc antibody. ABI1-Myc protein level gradually decreased with increasing PUB13-Flag (Fig. 4e, left). However, when the protoplasts were not treated with ABA, ABI1-Myc protein level was not obviously changed (Fig. 4e, middle). We also co-transfected *Pro35S:ABI1-Myc* plasmids and a mutated form *PYR1* plasmids plus increasing amount of *Pro35S:PUB13-Flag* plasmids into protoplasts. The mutated PYR1^P88S^ does not interact with ABI1 in the presence of ABA^7^. We did not observe a clear reduction of ABI1-Myc protein in the assay (Fig. 4e, right). These results suggest that PUB13 may promote ABI1-Myc degradation depending on both the presence of ABA and interaction with PYR1 in transgenic *Pro35S:PYR1-Flag Arabidopsis* cells. Although ABI1 and ABA-bound PYR1 form a stable complex, PYR1-Flag was not degraded by PUB13 in the assay (Fig. 4e). However, PYR1 must be degraded by other E3 ligases because PYR1 degradation also depends on the 26S proteasome (Supplementary Fig. 6). Consistently, PYR1/PYL4 and PYL8 have been shown to be degraded by a single subunit RING-type E3 ubiquitin ligase RSL1 (RING FINGER OF SEED LONGEVITY1) and a DDA1 (DET1-, DDB1-ASSOCIATED1) E3 ligase, respectively^36^,^37^.

To determine whether ABI1 can be ubiquitinated in plant cells, we used a P62-agarose matrix that is capable of binding ubiquitinated proteins to enrich the ubiquitinated proteins from two independent transgenic seedlings stably expressing *Pro35S:ABI1-Myc* or from wild-type plants as a negative control. The bound proteins were used for immunoblotting analysis with anti-Myc antibody. As shown in Fig. 4f, the ladder-like protein pattern was detected in the enriched proteins from two transgenic plants but not from wild-type plants. This result indicates that ABI1 can be ubiquitinated in plant cells.

### ABA promotes ABI1 degradation in plants

**Figure 5:**
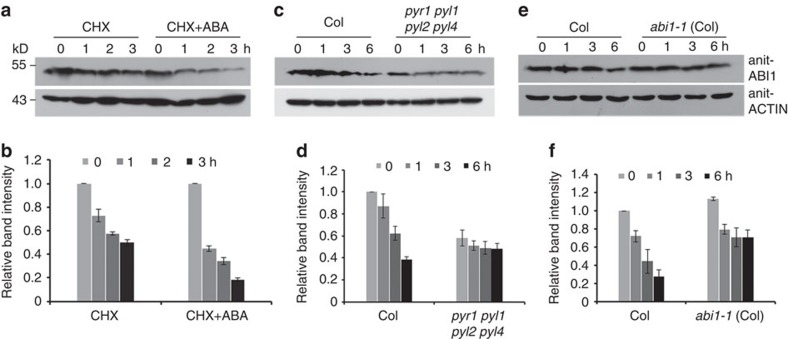
ABI1 degradation is promoted by ABA or ABA receptors in plants, and delayed by ABI1-1 dominant mutation. (**a**) ABA promotes ABI1 degradation. The 7-day-old wild type seedlings were treated with 100 μM CHX or 100 μM CHX plus 50 μM ABA for different times. At each time point, proteins were extracted and used for immunoblotting analysis. ACTIN was used as a loading control. (**b**) Quantitative analysis of the signal intensity in **a**. The abundance of ABI1 at the 0 h (CHX, CHX+ABA) was set to 1 as a reference for calculating relative abundance of various time point. Error bars are means±s.e.m. (*n*=3 independent experiments). (**c**) ABI1 protein is more stabilized in *pyr1 pyl1 pyl2 pyl4* quadruple mutant than in the wild type. The total extracted proteins were extracted from 7-day-old seedlings treated with 100 μM CHX for different times and used for immunoblotting analysis with anti-ABI1 antibody. ACTIN was used as a loading control. (**d**) Quantitative analysis of the signal intensity in **e**. The abundance of ABI1 at the Col 0 h was set to 1 as a reference for calculating relative abundance of various time point. Error bars are means±s.e.m. (*n*=3 independent experiments). (**e**) ABI1 protein is more stable in *abi1-1* than in the wild type. The total extracted proteins from the 7-day-old wild type or *abi1-1* (Col) seedlings treated with 100 μM CHX for different times were used for immunoblotting analysis with anti-ABI1 antibody. ACTIN was used as a loading control. (**f**) Quantitative analysis of the signal intensity in **g**. The abundance of ABI1 at the Col 0 h was set to 1 as a reference for calculating relative abundance of various time point. Error bars are means±s.e.m. (*n*=3 independent experiments).

As ABA is absolutely required for the interaction of ABI1 and PYR1 in order for PUB12/13 to ubiquitinate ABI1 in the *in vitro* assay (Fig. 3a,b), we asked whether ABA influences ABI1 degradation in plants. The wild-type seedlings were treated with 100 μM CHX or treated with 100 μM CHX plus 50 μM ABA for 0, 1, 2 and 3 h, and then the total proteins were used for immunoblotting with anti-ABI1 antibody. ABA treatment reduced the ABI1 protein level more than the control treatment (Fig. 5a,b). We also purified ABI1-His from *E. coli* and added it to total proteins extracted either from the wild-type plants or from an ABA-deficient mutant *aba2-21* (containing <10% ABA of the wild type)^38^ in presence of ATP. ABI1-His was more degraded in the extracts from the wild type than *aba2-21* in these cell-free 26S proteasome assays (Supplementary Fig. 7). These results suggest that ABI1 degradation is promoted by ABA.

### ABA receptors are required for ABI1 degradation

If the interaction of ABI1 and PYLs is a prerequisite for PUB12/13-mediated ubiquitination of ABI1, then we hypothesized that reduced degradation of ABI1 would be observed in PYL mutants. To this end, total proteins were extracted from PYL quadruple mutants (*pyr1 pyl1 pyl2 pyl4*)^7^ or the wild-type plants treated with 100 μM CHX for 0, 1, 3 and 6 h, and used for immunoblotting with anti-ABI1 antibody. As shown in Fig. 5c,d, ABI1 protein level was lower in the PYL quadruple mutant than the wild type, but the degradation of ABI1 was much slower in the quadruple mutant than in the wild type. Moreover, ABI1 protein level induced by ABA was much less in the quadruple mutant than in the wild type (Supplementary Fig. 8a). We further performed a cell-free protein degradation assay by combining total proteins extracted from the wild type or the quadruple mutant with ABI1-His protein purified from *E. coli* in presence of ATP. The results showed that the degradation of ABI1-His was slower in protein extraction from the quadruple mutant than from the wild type (Supplementary Fig. 8b). On the basis of these results we conclude that ABI1 degradation requires ABA receptors.

As the mutation in ABI1-1 blocks its interaction with ABA receptors, ABI1-1 is not ubiquitinated in the *in vitro* assay (Fig. 3a,b). We next examined ABI1-1 stability in plant cells by determining the ABI1 level with anti-ABI1 antibody. Immunoblotting analysis indicated that ABI1 was accumulated more under normal growth condition, but less with ABA treatment in *abi1-1* mutant than the wild type (Supplementary Fig. 9a). After seedlings were treated with 100 μM CHX for different times, ABI1 protein level was checked. ABI1 protein was gradually reduced in the wild type, but only reduced to a certain level in *abi1-1* (Col) as time went on (Fig. 5e,f). In addition, we transiently transfected ABI1-1-Myc or ABI1-Myc plasmids, respectively into *Arabidopsis* protoplasts. After the protoplasts were cultured for 14 h, total proteins were extracted from these protoplasts and used for the cell-free protein degradation assay. The degradation of ABI1 wild-type protein was greatly enhanced compared with mutated ABI1-1 in presence of ATP (Supplementary Fig. 9b). These results imply that the reduced ABI1-1 degradation is likely due to its failed interaction with ABA-bound PYLs.

### PUB12/13 are involved in the ABA signalling pathway

**Figure 6:**
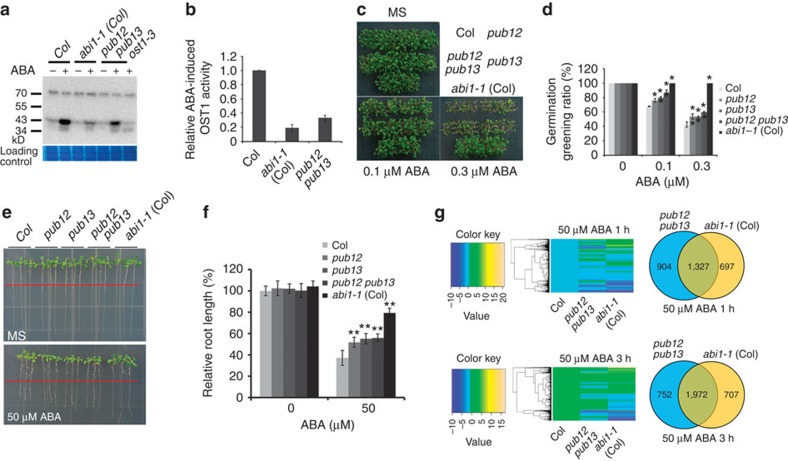
**The** ***pub12 pub13*** **mutant exhibits reduced sensitivity to ABA.** (**a**) *pub12 pub13* mutant reduces ABA-activated OST1 activity in an in-gel assay. Total proteins were isolated from 2-week-old seedlings treated with 50 μM ABA or without ABA for 30 min, and separated by SDS–PAGE gel containing 0.1 mg ml^−1^ MBP substrate. RUBISCO was used as a loading control. Three independent experiments were done with similar results. (**b**) Quantitative analysis of the band intensity in **a**. The band intensity of OST1 in the wild type with ABA treatment was set to 1, which was compared with the band intensity from *abi1-1* (Col) or *pub12 pub13* with ABA treatment. Error bars are means±s.e.m. (*n*=3 independent experiments). (**c**) Seed germination greening analysis of *pub12*, *pub13*, *pub12 pub13*, *abi1-1* (Col) and the wild-type (Columbia, Col) seeds on MS medium or MS medium supplemented with 0.1 and 0.3 μM ABA. (**d**) Seed germination greening ratio in **b**. Three independent experiments were done, each with three replicates. Error bars are means±s.d., *n*=3 (**P<0.01, Student's *t*-test). (**e**) Reduced sensitivity to ABA in root growth of *pub12*, *pub13*, and *pub12 pub13* mutants compared with the wild type. *abi1-1* (Col, *abi1-1* in Columbia accession) was included as a positive control. (**f**) Statistical analysis of ABA-inhibited root growth in **e**. Root length is relative to the control (without ABA). Three independent experiments were conducted, each with three replicates. Error bars mean±s.d., *n*=3 (**P<0.01, Student's *t*-test). (**g**) RNA-seq analyses of the ABA-responsive genes in *pub12 pub13*, *abi1-1* (Col) and the wild type (Col). Data are obtained from two independent experiments. The genes that are induced by 50 μM ABA at 1 h or 3 h were selected. The heat map was drew according to the expression levels in the wild type, *pub12 pub13* and *abi1-1* (Col) at ABA treatment for 1 h or 3 h (See Methods for detail). The overlapped numbers (right) between *pub12 pub13* and *abi1-1* (Col) are the ABA-induced genes that have lower expression levels in both *pub12 pub13* and *abi1-1* than in the wild type at ABA treatment for 1 or 3 h.

Because PUB12/13 target ABI1 for its degradation and because the *pub12 pub13* mutant greatly reduces ABI1 degradation compared with the wild type, we speculated that the *pub12 pub13* mutant would reduce the ABA response. SnRK2.2/2.3/2.6 are specially inhibited by clade A PP2Cs^6^,^7^ and ABA-activated OST1/SnRK2.6 is one of the most important outputs in ABA signalling. An in-gel assay using total proteins extracted from seedlings treated with ABA indicated that protein kinase activity corresponding to OST1 (OST1 shows the highest activity among SnRK2.2, SnRK2.3 and SnRK2.6/OST1) was lower in *pub12 pub13* mutant than in the wild type, but higher than in the *abi1-1* (Col) (Fig. 6a,b). The *ost1-3* mutant was used as a negative control. These results suggest that the reduced OST1 kinase activity results from increased activity of PP2Cs, most likely due to accumulation of ABI1 in the *pub12 pub13* mutant. As expected, *pub12* and *pub13* were more resistant to ABA than wild type when cotyledon greening after seed germination was examined (cotyledons become green after seed germination; Fig. 6c,d). The *pub12 pub13* double mutant showed an enhanced ABA-insensitivity phenotype in cotyledon greening relative to *pub12* or *pub13* (Fig. 6c,d). *pub12*, *pub13* and *pub12 pub13* were also more resistant to inhibition of root growth by ABA than the wild type (Fig. 6e,f). However, the ABA-resistant cotyledon greening and root growth phenotypes were much weaker in the *pub12 pub13* mutant than in *abi1-1* (Col)^4^,^5^,^35^. A recent study suggests that ABI1 is a negative mediator in plant freezing tolerance^39^. Consistently, *pub12 pub13* seedlings showed increased sensitivity and ion leakage to freezing stress under both non-acclimated and cold-acclimated conditions compared with the wild type (Supplementary Fig. 10).

As *pub12 pub13* mutant accumulates more ABI1 protein than the wild type, we expected that *pub12 pub1*3 could regulate the expression of some common genes as *abi1-1* (Col) under ABA treatment conditions. Ten-day-old seedlings were treated with 50 μM ABA for 0, 1 and 3 h. Total RNAs were isolated and used for RNA-deep sequencing on Illumina Hiseq platform. The 125 bp trimmed paired-end reads with high quality were generated and mapped to the *Arabidopsis* genome (TAIR10) using TopHat ( http://tophat.cbcb.umd.edu/) with default settings^40^. RNA-seq data were collected from two independent experiments (each sample with 2.0 G clean data) and differential gene expression analysis was performed using Cufflinks ( http://cufflinks.cbcb.umd.edu/)^41^. These analyses identified 3,580 and 4,225 genes that were significantly induced by 50 μM ABA at 1 h and 3 h, respectively, in the wild type (Supplementary Data 2 and 3). We then compared the expression levels of these ABA-induced genes in the wild type with those in *pub12 pub13* and *abi1-1* (Col). Among 3580 ABA-induced genes at 1 h, the expression levels of 2,237 genes were lower in *pub12 pub13*, and 2,024 genes lower in *abi1-1* (Col) than the wild type (Fig. 6g; Supplementary Data 2 and 3). The expression levels of 1,327 genes were lower in both *pub12 pub13* and *abi1-1* (Col) than in the wild type. Similarly, among 4,225 ABA-induced genes at 3 h, 2,724 genes in *pub12 pub13* and 2,679 genes in *abi1-1* (Col) were downregulated, and 1,972 genes were downregulated in both *pub12 pub13* and *abi1-1* (Col) compared with the wild type (Fig. 6g). The expression levels of ABA-induced marker genes such as *RD29A* (*AT5G52310*), *KIN1* (*AT5G01520*), *ABI1* (*AT4G26080*), *ABI2* (*AT1G17550*), *HAB1* (*AT1G72770*), *HAB2* (*AT1G17550*) in *pub12 pub13* were higher than in *abi1-1* (Col), but lower than in the wild type (Supplementary Data 2 and 3). Heat map analysis indicates that the expression of ABA-induced genes has a strong correlation between *pub12 pub13* and *abi1-1* (Col) (Fig. 6g), which implies that *pub12 pub13* mutant reduces ABA signalling likely through promoting accumulation of ABI1 protein.

**Figure 7:**
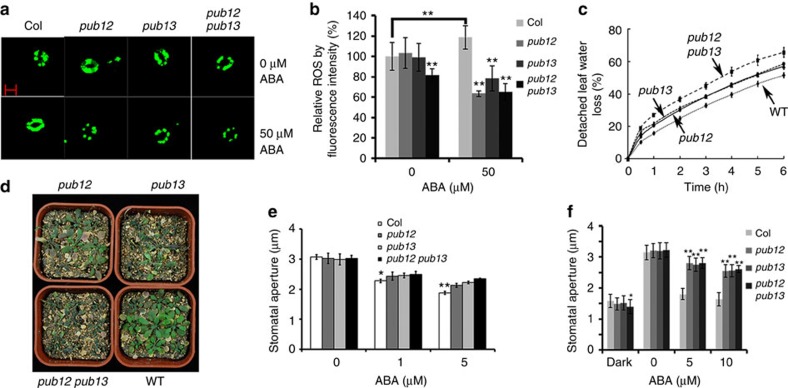
The ***pub12 pub13*** **mutant shows reduced ABA sensitivity in stomatal movement.** (**a**) ABA-induced H_2_O_2_ accumulation in guard cells is lower in the *pub12 pub13* mutant than in the wild type. H_2_O_2_ accumulation was assessed by H_2_DCF-DA staining following treatment with 0 or 50 μM ABA for 5 min. Scale bar, 10 μm. (**b**) The relative fluorescence intensity of guard cells in **a**. Fluorescence intensity is relative to the wild type without ABA treatment. Values are means±s.d. of three replicates (30 stomata from one seedling in each replicate) from one representative experiment; three independent experiments were done with similar results (**P<0.01, Student's *t*-test). (**c**) Water loss from detached leaves is greater in *pub12*, *pub13* and *pub12 pub13* than in the wild type. Three independent experiments were done with similar results. Values are means±s.d. of three replicates (40 leaves from one pot were measured per replicate) from one representative experiment. (**d**) *pub12*, *pub13* and *pub12 pub13* mutants are more sensitive to drought stress than the wild type grown in soil. Water was withheld from 19-day-old seedlings growing in soil; after 10 days without water, the seedlings were photographed. (**e**) *pub12 pub13* mutations impair ABA-induced stomatal closure. Leaf epidermal peels were treated with MES buffer for 2 h under strong light to fully open stomata. After the peels were incubated with different concentrations of ABA for 2 h, stomatal apertures were measured with Image J. Values are means±s.d. of three replicates (120–150 stomata from one seedling in each replicate) from one representative experiment; three independent experiments were done with similar results (**P<0.01, *P<0.05, Student's *t*-test). (**f**) The *pub12 pub13* mutant has impaired ABA-inhibited stomatal opening. Four-week-old seedlings were kept in darkness for 24 h. Different concentrations of ABA were then added, and seedlings were kept under strong light for 3 h before stomatal apertures were measured. Values are means±s.d. of three replicates (120–150 stomata from one seedling in each replicate) from one representative experiment; three independent experiments were done with similar results (**P<0.01, *P<0.05, Student's *t*-test).

ABA promotes the production of H_2_O_2_ in guard cells^42^,^43^. In the ABA signalling pathway, ABI1 acts as a negative factor upstream of H_2_O_2_ to mediate stomatal movement^44^. The *abi1-1* mutation greatly reduces H_2_O_2_ production in guard cells^44^. We expected that the mutations in *pub12 pub13* would cause ABI1 to accumulate, which would result in the reduced accumulation of H_2_O_2_ in guard cells. In the absence of ABA, the guard cells of *pub12 pub13* produced less H_2_O_2_ than those of the wild type or the *pub12* or *pub13* mutant (Fig. 7a,b). ABA treatment significantly increased H_2_O_2_ production in the guard cells of the wild type but significantly decreased H_2_O_2_ production in guard cells of *pub12*, *pub13* or *pub12 pub13* (Fig. 7a,b). These results indicate that the decreased H_2_O_2_ production following ABA treatment in *pub12 pub13* guard cells is likely due to ABI1 accumulation. In a detached-leaf water loss assay, *pub12* and *pub13* lost more water than the wild type, and water loss was greater in the *pub12 pub13* double mutant than in *pub12* or *pub13* single mutants (Fig. 7c). In soil, the *pub12*, *pub13* and *pub12 pub13* mutants lost more water and were more sensitive to drought stress than the wild type (Fig. 7d). Using isolated epidermal peels, we found that ABA-induced stomatal closure (Fig. 7e) and ABA-inhibited stomatal opening (Fig. 7f) were impaired in *pub12*, *pub13* and *pub12 pub13* mutants. These results indicate that PUB12 and PUB13 are involved in ABA-mediated stomatal movement.

### *abi1-3* recovers ABA-insensitivity of *pub12 pub13*

**Figure 8:**
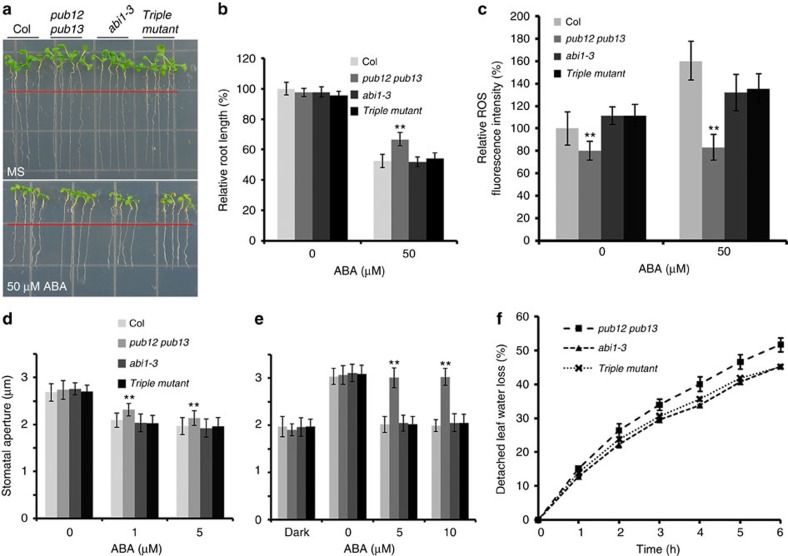
***abi1-3*** **loss-of-function mutant recovers the ABA-insensitive phenotypes of** ***pub12 pub13***. (**a**) The *abi1-3 pub12 pub13* triple mutant is similar to *abi1-3* or wild type, but more sensitive to ABA than *pub12 pub13* mutant. (**b**) Statistical analysis of ABA-inhibited root growth in **a**. Root length is relative to the control (without ABA). Three independent experiments were conducted, each with three replicates. Values are means±s.d., *n*=3 (**P<0.01, Student's *t*-test). (**c**) ABA-induced H_2_O_2_ accumulation in guard cells is comparable among *abi1-3*, *abi1-3 pub12 pub13* triple mutant and the wild type that produces more H_2_O_2_ than the *pub12 pub13* double mutant. H_2_O_2_ accumulation was assessed by H2DCF-DA staining following treatment with 0 or 50 μM ABA for 5 min. Fluorescence intensity is relative to the wild type without ABA treatment. Values are means±s.d. of three replicates (30 stomata from one seedling in each replicate) from one representative experiment; three independent experiments were done with similar results (**P<0.01, Student's *t*-test). (**d**) The *abi1-3 pub12 pub13* triple mutant recovers the impaired ABA-induced stomatal closure. The same treatment was done as in Fig. 7e. Values are means±s.d. of three replicates (120–150 stomata from one seedling in each replicate) from one representative experiment; three independent experiments were done with similar results (**P<0.01, Student's *t*-test). (**e**) The *abi1-3 pub12 pub13* triple mutant recovers the impaired ABA-inhibited stomatal opening of *pub12 pub13*. The same treatment was done as in Fig. 7f. Values are means±s.d. of three replicates (120–150 stomata from one seedling in each replicate) from one representative experiment; three independent experiments were done with similar results (**P<0.01, Student's *t*-test). (**f**) Water loss from detached leaves is similar in the *abi1-3 pub12 pub13* triple mutant and *abi1-3* that lose less water than *pub12 pub13*. Values are means±s.d. of three replicates (40 leaves from one pot were measured per replicate) from one representative experiment.

The above results suggest that PUB12/13 target ABI1 for its degradation. If this is the case, genetically, ABI1 should act downstream of PUB12/13, and *abi1* loss-of-function mutant should block ABA-insensitive phenotypes of *pub12 pub13* mutant. In order to test this hypothesis, we introduced the *abi1-3* loss-of-function allele into *pub12 pub13* mutant by crossing *abi1-3* (a T-DNA insertion mutant, Supplementary Fig. 1 for ABI1 protein level)^45^ with *pub12 pub13* and tested the ABA response of the *abi1-3 pub12 pub13* triple mutant. Previous studies show that *abi1* loss-of-function mutant does not show any apparent ABA phenotype compared with the wild type as these PP2Cs are redundant in ABA signalling^45^,^46^. We first compared the root growth of the *abi1-3 pub12 pub13* triple mutant with the *pub12 pub13* double mutant and *abi1-3* with ABA treatment. As shown in Fig. 8a,b, the *abi1-3 pub12 pub13* triple mutant showed similar root growth phenotype as *abi1-3* or the wild type with ABA treatment. The root growth of *pub12 pub13* was more resistant to ABA than the triple mutant, *abi1-3* or the wild type. We further compared the ROS production. *abi1-3 pub12 pub13* triple mutants produced similar amount of ROS as *abi1-3*, but significantly more than *pub12 pub13* without or with ABA treatment (Fig. 8c). Furthermore, *abi1-3 pub12 pub13* triple mutant exhibited similar ABA-induced stomatal closure (Fig. 8d) and ABA-inhibited stomatal opening (Fig. 8e) as *abi1-3* or the wild type, indicating that ABI1 loss function recovers the impairment of ABA-regulated stomatal movement in *pub12 pub13*. In detached-leaf water loss, *abi1-3 pub12 pub13* triple mutant lost similar water as *abi1-3*, but less than *pub12 pub13* (Fig. 8f). All these genetic data indicate that PUB12/13 act upstream of ABI1 to modulate ABA response.

## Discussion

**Figure 9:**
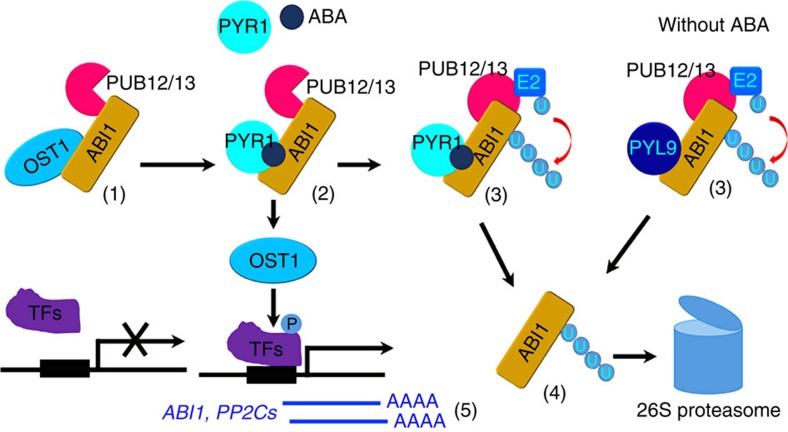
A proposed model for ABI1 degradation. (1) ABI1 interacts with and inhibits OST1. ABI1 can also interact with PUB12/13. (2) After ABA-bound PYR1 interacts with ABI1, protein kinases such as OST1 are released and activated to phosphorylate downstream targets including transcriptional factors (TFs) and SLAC1 in guard cells. (3) PUB12/13 are able to ubiquitinate ABI1 likely because of the conformational change of ABI1 after it interacts with PYR1 (with ABA) or PYL9 (with and without ABA). (4) The ubiquitinated ABI1 is degraded by 26S proteasomes. (5) The transcripts of *ABI1* and other related *PP2Cs* are induced by ABA signaling.

PP2Cs are key repressors in the ABA signalling pathway. The ABA receptors PYLs bind to ABA, which allows the capture of PP2C proteins and the inhibition of PP2C activity. The entire ABA signalling pathway in *Arabidopsis* can be reconstituted *in vitro* by co-expression of ABA signalling core components including PYLs, PP2Cs, SnRKs and ABF2 (ref. 47), suggesting that the PP2C inhibition by ABA-bound PYLs is sufficient to activate SnRKs. Previous studies indicate that *ABI1* is upregulated at transcriptional level by ABA in a negative feedback regulatory loop^48^. In this study, we found that when interacting with ABA receptors, ABI1 is degraded by the 26S proteasome pathway, which consequently enhances ABA signalling (Fig. 9). These results suggest that both the inhibition and degradation of ABI1 are important for activating ABA signalling *in vivo*. Our results suggest that PUB12/13 can interact with ABI1 but can ubiquitinate ABI1 only when ABI1 interacts with PLYs both with ABA (such as with PYR1) and without ABA (such as with PYL4/9) in the *in vitro* assays (Fig. 3a,b). However, ABA apparently promotes the degradation of ABI1 (Fig. 5a; Supplementary Fig. 7). It is possible that interaction of ABI1 with PYLs changes the conformation of ABI1, which may create a suitable surface for ubiquitin transfer.

Although the degradation of ABI1 in a PYL quadruple mutant is largely reduced (Fig. 5), ABI1 protein level is lower in the PYL quadruple mutant than in the wild type under both ABA treatment and control treatment (that is, normal growth condition), consistent with previous studies that show ABA signalling is reduced by PYL mutations^6^. Interestingly, ABI1 protein is even higher in *abi1-1* mutant than in the wild type with no ABA treatment (under normal growth condition), but lower with ABA treatment. As the ABI1-1 mutation is hypermorphic, ABA signalling is greatly reduced in the *abi1-1* mutant. Because the transcripts of *ABI1* are at least not more in *abi1-1* than in the wild type (Fig. 4b), the higher accumulation of ABI1 protein in *abi1-1* than the wild type suggests that ABI1-1 protein is more stable in *abi1-1* than in the wild type under normal condition. However, under ABA treatment, ABI1 level is much less in *abi1-1* than the wild type because ABI1-1 cannot be inhibited by ABA receptors, which blocks ABA signalling and reduces the *ABI1* transcripts even if ABI1-1 degradation is reduced (Figs 4b and 5e). These results suggest that when ABA signalling is strongly stimulated, a higher ABI1 level through transcriptional control is required to attenuate this signalling. ABI1 level is dynamically modulated at both transcriptional (upregulated by ABA) and protein level (downregulated by ABA).

The *ABI1* transcript level is reduced but the ABI1 protein level is higher in the *pub12 pub13* mutant compared with the wild type after both ABA and control treatments. The degradation of ABI1 protein is slower in *pub12 pub13* mutant than in the wild type. These results suggest that ABI1 protein stability is mediated by PUB12/13. We propose that ABI1 can be ubiquitinated by PUB12/13 and targeted to the proteasome for degradation after it interacts with ABA receptors PYL4 and PYL9 without ABA to maintain the homeostasis of ABI1 so that cells can efficiently relieve the repression mediated by ABI1. When ABA is accumulated under stress conditions, ubiquitination and degradation of ABI1 would be promoted to enhance ABA signalling/response.

In yeast two-hybrid assays, we also found that ABI1 interacts with other E3 ligases. Although we failed to detect their interaction in plant cells, we cannot exclude the roles of these E3s for mediating ABI1 degradation under specific conditions or in special tissues/cells. However, our genetic analyses (Fig. 8) indicate that PUB12/13 are likely major modulators of ABI1 degradation in *Arabidopsis* as introducing the *abi1-3* null mutation could completely recover the ABA-insensitive phenotypes of the *pub12 pub13* mutant. Although ABI1 protein level is much higher in the *pub12 pub13* mutant compared with the wild type and *abi1-1* in both ABA treated and non-treated conditions, the ABA responses of *pub12 pub13* mutant are stronger than those of *abi1-1*, but weaker than those of the wild type (Fig. 6). The stronger ABA-insensitive phenotypes of *abi1-1* (Col) mutants may be caused by both the absence of PYL inhibition and by the reduced degradation of ABI1-1 protein (Fig. 5c,d). However, in the *pub12 pub13* mutant, ABI1 protein can still be inhibited by ABA receptors even if ABI1 is accumulated to a higher level than in *abi1-1*. Compared with the wild type, *pub12 pub13* mutant is more resistant to ABA as it accumulates more ABI1 protein. We did not observe any difference in ABA phenotypes between PUB13-overexpressing plants and the wild type (Supplementary Fig. 11), which is consistent with the observations that *abi1-3* knockout plants and wild-type plants do not clearly differ in ABA response and that PP2C triple mutants constitutively respond to ABA^46^.

To date, nine PP2Cs have been found to be involved in the ABA signalling pathway^49^. Here, we found that PUB12/13 specifically target ABI1 for its degradation, suggesting that other PP2Cs may also be regulated by different E3 ligases. For some ABA response phenotypes, these PP2Cs seem to be functionally redundant in plant cells but they probably have distinct roles in different tissues. In guard cells, for example, ABI1 preferentially interacts with and inhibits OST1 and acts upstream of H_2_O_2_, while ABI2 interacts with and inhibits GHR1 and acts downstream of H_2_O_2_ to control stomatal movement^10^,^44^. PP2Cs also differ in their interaction affinities with PYLs^49^. The combinations of different PP2Cs and PYLs, and the degradation of PP2Cs and PYLs by different E3 ligases may finely modulate ABA signalling^37^. Because PUB12/13 also target FLS2 for its turnover in immune signalling^29^, we speculate that PUB12 and PUB13 may link innate immune and ABA signalling. Recent studies indicate that the ABA signalling pathway integrates with the immune signalling pathway in plant responses to drought stress and pathogen attacks^50^,^51^. The molecular mechanisms governing such integration require further investigation in the future.

The proposed ABA signalling-regulation module (Fig. 9) is very similar to that for GA signalling, in which DELLA proteins are key repressors^3^,^52^. The interaction of the GA receptor GID1 with DELLA proteins are promoted by the binding of GA to GID1. The GID1-GA-DELLA complex facilitates the interaction of the DELLA C terminus with the F-box protein GID2-based SCF^GID2^ complex in rice (SLEEPY 1 [SLY1]-based SCF^SLY1^ in *Arabidopsis*), and DELLAs are in turn ubiquitinated and degraded through the 26S proteasome pathway^3^,^52^. The GID1-GA-DELLA complex would also reduce the availability of DELLA for interacting with and inhibiting its target transcriptional factors^53^,^54^. Like ABA and GA signalling, the signalling for other phytohormones such as auxin, jasmonate and strigolactone also follows a ‘Relief of Repression' module that degrades the negative regulators via receptor-/ SCF-26S proteasome-mediated proteolysis^3^,^55^,^56^. These results suggest that plants have evolved similar regulatory mechanisms in hormone signalling so as to quickly respond to environmental challenges under natural conditions.

## Methods

### Plant materials and growth conditions.

*Arabidopsis thaliana* (Col-0 accession) seeds were sown on MS medium containing 2% sucrose and 0.8% agar. At 5–7 days after germination, seedlings were transferred to soil and grown under short-day (12-h light/12-h dark) or long-day (16-h light/8-h dark) conditions in a growth room at 20–22 °C. The T-DNA insertion mutants used in this study were *pub13* (salk_093164) and *pub12* (wiscdslox497_01). For overexpression transgenic plants, the cDNAs of *ABI1*, *PUB12* and *PUB13* were amplified and cloned into the pCAMBIA1300 vector under the *35S* promoter. The correct clones were transformed into *Agrobacterium tumefaciens* strain GV3101 and transferred into *Arabidopsis* plants (wild type and the *pub12 pub13* double mutant) by floral dip method^57^. Twenty T3 homozygous transgenic lines were screened, and at least two lines were used for experiments. The primers used for identification of the mutations and for construction of transgenic plants are listed in Supplementary Table 1.

### Drought-related phenotype analyses

For a water loss assay with detached leaves, rosette leaves were cut from Col-0, *abi1-3, pub13*, *pub12*, *pub12 pub13*, *abi1-3 pub12 pub13* plants grown in soil under normal short-day conditions in a growth room. The detached leaves were weighed, placed on a piece of weighing paper in a growth room (20 °C and 75% humidity), and periodically weighed every hour for at least 6 h. Water loss was expressed as a percentage of the original fresh weight of the detached leaves. The experiment was independently repeated twice.

For stomatal aperture measurement, epidermal strips were peeled from rosette leaves of 4-week-old seedlings. The chlorophyll on the epidermal strips was removed with a writing brush. The epidermal strips were then immersed in opening solution MES buffer (10 mM MES-KOH (pH 6.15), 10 mM KCl and 50 μM CaCl_2_) under light (90 μmol m^−2^ s^−1^) for 2 h at 22 °C. The treated epidermal strips were then transferred to MES buffer containing 0, 1 or 5 μM ABA. After incubation for 2 h in light, the epidermal strips were photographed with an OLYMPUS BX53 microscope and were measured with Image J 1.47V software. For an assay assessing ABA inhibition of stomatal opening under light, 4-week-old plants were cultured in darkness for 24 h to make the stomata close. Then, epidermal strips were quickly peeled and immersed under light (90 μmol m^−2^ s^−1^) in MES buffer containing 0, 5 or 10 μM ABA for 3 h at 22 °C before stomatal apertures were photographed with an OLYMPUS BX53 microscope and measured with Image J 1.47V software. For each combination of genotype and sampling time, 120–150 stomata were measured, and three independent experiments were done.

For the drought phenotype assay, 5-day-old seedlings were transferred to soil and grown under short-day conditions. When the seedlings were 19-day old, water was withheld. After the water had been withheld for 10 days, the seedlings were photographed.

### Determination of ROS production

The production of H_2_O_2_ was detected by an H2DCF-DA staining assay as described previously^10^. Epidermal strips were taken from rosette leaves of 4-week-old seedlings. The epidermal strips without mesophyll cells were soaked in MES buffer (10 mM MES-KOH (pH 6.15), 10 mM KCl, and 50 μM CaCl_2_) for 3 h at 22 °C to eliminate the excess ROS generated during the operation. Then, 50 μM H2DCF-DA (Sigma-Aldrich, cat. no. D6883) was added to the buffer. The samples were cultured in darkness for 20 min and then washed five times with ddH_2_O to remove the excess H2DCF-DA. Finally, the epidermal strips were stored in MES buffer. After 50 μM ABA was added to the buffer for 5 min, the fluorescence in guard cells was detected with a confocal microscope (Zeiss LSM 510 META). The fluorescence intensities were analysed using AxioVisionRel. 4.8 software. About 30 guard cells were assessed per sample, and the experiment was independently performed three times.

### Gene expression analysis by quantitative RT–PCR

The seedlings were treated with 50 μM ABA for different times as indicated. Total RNAs were extracted from these seedlings with TRIzol reagent (Life Technologies, cat. no. 15596-018). A 4-μg quantity of DNase I-treated total RNAs was used as template for first-strand cDNA synthesis by M-MLV reverse transcriptase (Promega, cat. no. M170A). cDNAs were diluted 10 times with ddH_2_O, and 2 μl was used for PCR. quantitative PCR with reverse transcription (qRT–PCR) was performed with SYBR premix ExTaq (TaKaRa, cat. no. RR820A) and with gene-specific primers and the internal control (Actin4). qRT–PCR was performed with a 7300 Real-Time PCR system. The reaction conditions included 40 cycles at 95 °C for 5 min, 95 °C for 15 s and 60 °C for 34 s. The primers used for qRT–PCR are listed in Supplementary Table 1.

### GUS staining

The promoters of *PUB12* (−2,332 to −1) and *PUB13* (−2,139 to −1) were fused to pCAMBIA1391 vector by *Pst* I and *EcoR* I sites. The cloned constructs were transformed into *Agrobacterium* GV3101 strain, and transferred into wild type *Arabidopsis* by floral dip method^57^. The transgenic seedlings of T2 plants were used for GUS staining. The primers used for *ProPUB12:GUS* and *ProPUB13:GUS* were listed in Supplementary Table 1.

### *In vitro* ubiquitination assay

The *in vitro* ubiquitination assay was performed as described previously^29^. In brief, *PUB12*, *PUB13*, *PYR1* and *UBC8* (E2) were separately cloned into the pGEX-4T-1 vector, ABI1 was fused into the pET-28a vector. The recombinant proteins were extracted from *E. coli* strain BL21 (DE3). The primers used for this assay are listed in Supplementary Table 1. The fusion proteins were purified with glutathione-sepharose and Ni-sepharose. A 250-ng quantity of wheat (*Triticum aestivum*) E1, 500 ng of purified E2-GST (UBC8), 1.25 μg of Flag-tagged ubiquitin (Boston Biochem, cat. no. U-120), 1 μg of purified PUB12/13-GST, 500 ng of ABI1-His substrate, 500 ng PYR1-GST and 5 μM ABA were added to 30 μl of ubiquitination reaction buffer (50 mM Tris-Cl pH 7.5, 2 mM ATP, 5 mM MgCl_2_, 2 mM DTT)^29^. After 2 h at 30 °C with oscillation in a thermomixer (Eppendorf), the reactions were stopped by adding 4 × SDS loading buffer; the samples were the boiled at 100 °C for 5 min. The products were electrophoresed on a 10% SDS–polyacrylamide gel electrophoresis (PAGE) gel and detected with anti-His and anti-Flag antibody by western blotting.

### Co-IP assays

For Co-IP experiments, protoplasts transformed with a *Pro35S:PUB13-Flag*, *Pro35S:PUB12-Flag*, *Pro35S:ABI1-Myc* construct, and others were incubated in 1 ml of W5 buffer (154 mM NaCl, 125 mM CaCl_2_, 5 mM KCl and 2 mM MES (pH 5.7)) for 14–16 h. The primers used to construct the vectors are listed in Supplementary Table 1. The protoplasts were then collected, lysed in 1 ml of protein extraction buffer (10 mM HEPEs (pH 7.5), 100 mM NaCl, 1 mM EDTA, 10% glycerol, 0.5% Triton X-100, protease inhibitor cocktail and 1 mM PMSF)^29^, and centrifuged at 12,000*g* for 10 min at 4 °C of the 1,000 μl of supernatant, 80 μl was reserved as input, and the remaining volume was incubated on an Anti-c-Myc-Agarose Affinity Gel (Sigma-Aldrich, cat. no. A7470) for 2 h at 4 °C. The beads were then washed with PBS (pH 7.5) 3–4 times. The immunoprecipitated proteins were analysed by immunoblotting analysis.

### Cell-free protein degradation assay

Cell-free protein degradation assay was performed as described with some modifications^58^. Wild-type and mutant (*aba2-21*) total proteins were extracted with native protein extraction buffer (50 mM Tris-MES (pH 8.0), 0.5 M sucrose, 1 mM MgCl_2_, 10 mM EDTA (pH 8.0), 5 mM DTT). For Fig. 1c, the extracted supernatants were divided into two equal parts with addition of 1 mM ATP or not, and the samples were cultured at 25 °C for different times. 4 × SDS loading buffer was added to stop reactions. The samples were boiled and then tested with anti-ABI1. For Supplementary Fig. 7, 200 ng purified proteins ABI1-His from *E. coli* strain BL21 (DE3) were incubated in 100 μl protein crude extraction (containing 500 μg total proteins) for each reaction with addition of 1 mM ATP, and cultured at 25 °C for different times. Anti-His antibody was used to detect ABI1-His proteins level by immunoblotting analysis.

### Firefly luciferase complementation imaging assay

The full-length cDNA sequence of PUB12, PUB13, PUB12 ARM, PUB13 ARM were fused to the N-terminal of pCAMBIA-nLUC, and ABI1 was fused to the C-terminal of pCAMBIA-cLUC vectors. The sequence of FLS2 was fused to the C-terminal of modified pCAMBIA-cLUC vectors (the sequence of cLUC was moved to the back of multiple cloning site). The constructed plasmid vectors were transformed into *Agrobacterium* strain GV3101. The positive clone was incubated in YEB liquid medium at 28 °C for 16 h. The bacteria were mixed at a properly final *A*_600_ (*A*_600_=1.5 for *ABI1*, and *A*_600_=0.5 for *PUB12/13* and *PUB12/13* ARM), then the bacteria were collected and resuspended in 2 ml of activity buffer (10 mM MES (pH 5.7), 10 mM MgCl_2_, 150 μM acetosyringone). After 2–5 h, the activity bacteria were injected into young *Nicotiana benthamiana* leaves. After 3 days, the abaxial sides of leaves were sprayed with 1 mM luciferin and then kept in the dark for 5 min. A CCD (charge-coupled device; 1300B, Roper) camera was used to capture the LUC signal at −110 °C. The exposure time was 15 min for cLUC-ABI1+PUB13 ARM-nLUC and cLUC-ABI1+PUB12 ARM-nLUC, and was 60 min for cLUC-ABI1+PUB13-nLUC and cLUC-ABI1+PUB12-nLUC. The primers used for this assay were listed in Supplementary Table 1.

### Purification of ubiquitinated proteins

Wild-type and two *Pro35S:ABI1-Myc* transgenic plants were grown in MS medium for 10 days and were then treated with 50 μM ABA for 12 h and 50 μM MG132 for 6 h. Total proteins were extracted with 1 ml of BI buffer (50 mM Tris-Cl, (pH 7.5), 20 mM NaCl, 0.1% NP-40 and 5 mM ATP) in a prechilled mortar. The following were added to the protein homogenates: 1 mM PMSF, 50 μM MG132, 10 nM Ub aldehyde (Sigma-Aldrich, cat. no. SRP6024), and 10 mM *N*-ethylmaleimide (Sigma-Aldrich, cat. no. E1271). After proteins were quantified, 2 mg of total proteins in a total volume of 2 ml was used or the assay. An 80 μl volume of protein supernatants was reserved as input. Other protein supernatants were incubated with 40 μl of prewashed p62-agarose (Enzo Life Sciences, cat. no. BML-UW9010-0500) in 2 ml of BI buffer at 4 °C. After 4 h, the agaroses were washed two times with BI buffer and once with BII buffer (supplemented with 200 mM NaCl in BI). Samples were boiled in 50 μl of 1 × SDS loading buffer for 5 min. The ubiquitinated proteins were separated by 10% SDS–PAGE gel, and anti-Myc antibody was used to detect ubiquitinated ABI1-Myc protein. ACTIN was used as an equal loading control.

### Yeast two-hybrid assay

To confirm the interaction between ABI1 and PUB12/13, and between ABI1 and other proteins, full-length *PUB12/13* or other genes and *ABI1* cDNA were separately fused into *pGBKT7* (binding domain, BD) and *pGADT7* (activation domain, AD) vectors. These plasmids were co-transformed into yeast strain AH109. Transformed yeast cells were separately sprayed onto 2D synthetic dropout medium (−Trp/−Leu) and 3D selective medium (−Trp/−Leu/−His), and incubated at 28 °C for 4–5 days. If the proteins in BD vector exhibited selfactivation, 30 mM 3-AT (3-amino-1, 2, 4-trazole) was added to suppress the selfactivation.

### High-throughput mRNA sequencing analyses

Ten-day-old seedlings grown on MS medium in the plastic plates under 23-h light/1-h dark at 22 °C were treated with 50 μM ABA for 0, 1 and 3 h. Total RNAs were extracted by RNeasy Plant Mini Kit (QIAGEN, cat. no. 74904) according to the kit instructions. Three microgram RNAs for each treatment were used for library construction and RNA-seq on Illumina Hiseq 2500 platform. The libraries were constructed using NEBNext Ultra RNA Library Prep Kit for Illumina (NEB, USA, cat. no. E7420L) following the instructions.

About 2.0-GB clean reads were generated for each sample (number of reads per sample and alignment statistics were listed in Supplementary Table 2). All reads were trimmed to 125-bp paired-end reads with high quality according to the base quality. (Original raw read length were about 250 bp, 125 bp paired-end clean reads were obtained by trimming the adapter sequence, if the ratio of bases with base quality *Q*_phred_<5 was >50% for one read, the paired-end reads were discard.) The trimmed reads were mapped to the genome of *A. thaliana* (TAIR10) using TopHat ( http://ccb.jhu.edu/software/tophat/index.shtml) with default settings^40^. Differential gene expression analysis was performed using Cufflinks ( http://cole-trapnell-lab.github.io/cufflinks/)^41^. Reads per kilobase of transcript per million reads mapped was used to indicate the gene expression level. Differentially expressed genes were selected by the comparison of gene expression levels of the control sample (treated 0 h with ABA) with treatment samples (1 or 3 h with ABA) for the wild type (Col), *pub12 pub13* and *abi1-1* (Col), respectively (using Student's *t*-test with *P*<0.01 and *q*<0.05). Genes significantly induced by ABA in the Col group were chosen for the comparison with expression levels of the treatment samples between different groups (Supplementary Data 2 and 3). Fold changes of the genes induced significantly by ABA treatment were compared with the control sample in each group. To compare the change levels of the treatment samples between different groups, we calculated relative expression level. Fold change of each gene in the treatment samples in *pub12 pub13* or *abi1-1* minus the fold change of the same gene in the treatment samples in Col was considered as the relative expression level of the gene in *pub12 pub13* or *abi1-1* comparing to Col. Thus positive or negative value of the relative expression level indicated the change level of the gene in *pub12 pub13* or *abi1-1* was higher or lower than the change level in Col. For purposes of presentation we multiplied the relative expression level by 5, and we considered multiplied relative expression level of less than −10 as −10. We then drew the heat maps based on the multiplied relative expression levels using heatmap.2 function in the gplots package in R. Complete linkage hierarchical clustering with Euclidean distance as a distance measure was used to sort the rows.

### In-gel kinase assay

In-gel kinase assay was performed as described^59^ with some modifications. In brief, total protein extracts were prepared from Col, *abi1-1* (Col) and *pub12 pub13* double mutant plants which were treated with or without 50 μM ABA for 30 min. Total proteins (40 μg) were separated by SDS–PAGE gel containing 0.1 mg ml^−1^ MBP substrate and then washed by washing buffer (1 mM DTT, 5 mM NaF, 0.1 mM Na_3_VO_4_, 0.5 mg ml^−1^ BSA, 0.1% Triton X-100, and 25 mM Tris-HCl, pH 7.5) for three times, 20 min each, to remove SDS. After removing SDS, the proteins were renatured with buffer containing 2 mM DTT, 5 mM NaF, 0.1 mM Na_3_VO_4_ and 25 mM Tris-HCl, pH 7.5, for 1, 12 (overnight) and 1 h at 4 °C. After 30 min of incubation with kinase reaction buffer (2 mM EGTA, 12 mM MgCl_2_, 1 mM DTT, 0.1 mM Na_3_VO_4_, and 25 mM HEPES-KOH, pH 7.5), the gel was incubated in 30 ml kinase reaction buffer supplemented with 60 μCi [γ-^32^P]ATP and 9 μl cold ATP (1 mM) at room temperature for 2 h and then washed with 5% TCA and 1% sodium pyrophosphate five times for 30 min each. Radioactivity was detected by Typhoon 9410 imager.

### Freezing tolerance and ion leakage assays

*Arabidopsis* plants were grown at 22 °C on MS medium containing 0.8% agar for 2 weeks. Then the seedlings were treated with or without cold acclimation at 4 °C for 4 days and were used to freezing assay in a freezing chamber (RuMED4001) as described in the previous study^39^. The programme was set to 1 °C and programmed to drop 1 °C per hour to experimental temperatures. After freezing treatment, the plants were put into 4 °C in the dark for 12 h and then transferred to normal conditions for 4 days and then counted the survival rates.

For ion leakage assay, seedlings were treated with freezing temperatures and placed into 15 ml tubes containing 5 ml deionized water (S0), which were shaken for 15 min and then detected S1. After detecting S1, the samples were boiled at 100 °C water for 15 min, shaken at 22 °C for 1 h, and then detected S2. Formula S1-S0/S2-S0 was used to calculate ion leakage.

### Immunoblotting analysis and quantitative analysis

Immunoblotting analysis and quantification were performed as described^60^. Total proteins were isolated from 7-day-old wild-type and mutant seedlings by protein extraction buffer (10 mM HEPEs, (pH 7.5), 100 mM NaCl, 1 mM EDTA, 10% glycerol, 0.5% Triton X-100 and protease inhibitor cocktail from Roche, PMSF from AMRESCO). Extracted proteins were quantified through BIO-RAD kit (#500-0006), added 4 × SDS loading buffer in the samples and boiled for 5 min. A total of 10% SDS–PAGE gels were used to separated proteins, and then were blotted onto nitrocellulose (MILLIPORE, cat. no. IPVH00010). The membranes were blocked in the blocking buffer (5% milk dissolved in 1 × PBS Tween-20 (PBST), adding 0.1% Tween-20 in 1 × PBS) at 25 °C for 2 h. The membranes were incubated overnight at 4 °C with a rabbit polyclonal antibody directed against full-length ABI1 protein or a mouse monoclonal antibody against ACTIN, both diluted 1/2,000 in the blocking buffer. Anti-ABI1 antibody was highly specific as no signal could be detected in *abi1-3* mutant as shown Supplementary Fig. 1. After washing three times in PBST for 10 min each, the membranes were incubated 2 h at room temperature with horseradish peroxidase-labelled goat anti-rabbit and goat anti-mouse, both diluted 1/10,000. Then washing three times in PBST for 10 min each, the membranes were incubated 3–5 min in ECL (29018904, GE). Then the signals were acquired by X-OMAT BT Film in darkroom. For quantitative analysis, the data were normalized by dividing the band intensity of ABI1 by the band intensity of ACTIN in each lane, firstly. Then the starting point (0 h) was set to 1, other points compare with it. Each experiment was independently repeated at least two times.

The ABI1 polyclonal antibodies were made by Beijing Protein Innovation Co., Ltd. (BPI). Briefly, a *Bam*HI/*Mfe*I fragment containing the full-length *ABI1* open reading frame with 6 × His was cloned into the *Bam*HI/*Eco*RI sites of the *pET-28a* vector. The fusion protein was expressed in *E. coli*, then purified and used as antigen to immunize rabbits for the production of polyclonal antiserum. Antigen affinity purified anti-ABI1 antibodies were used in immunoblots.

Other antibodies used for Immunoblotting assay were listed in Supplementary Table 3. All original immunoblots are provided in Supplementary Fig. 12.

### LC–MS/MS analysis

To detect the interaction proteins with ABI1, we performed LC–MS/MS assay using *ProABI1:ABI1-Flag* transgenic lines. Fifteen grams of transgenic plant seedlings were collected and grinded in liquid nitrogen. The grinded powder was dissolved in lysis buffer (50 mM Tris-HCl (pH 7.5), 150 mM NaCl, 10% Glycerol, 5 mM MgCl_2_, 0.5% NP-40; adding 1 mM PMSF protease inhibitor and 1 mM DTT before using). Then the samples were placed on ice for 10 min and centrifuged at 4,000*g*, 4 °C for 10 min. Supernatant was transferred to a new tube, and centrifuged at 12,000*g*, 4 °C for 20 min. Supernatant was transferred to a new tube containing 300 μl Flag-beads, incubated at 4 °C for 2.5 h with slowly shaking. After 500*g* centrifuge at 4 °C for 10 min, Flag-beads were collected and washed with lysis buffer for 3–5 times. A volume of 300 μl elution buffer (containing 100–500 μg ml^−1^ Flag peptide in PBS buffer) was added to Flag-beads, rotated at 4 ° for 1 h and repeated three times. All of the elution buffer (total about 900 μl) was collected together and concentrated with ultra-filtration column (Millipore, cat. no. UFC501024) to 50 μl as the final sample. The sample was separated in 10% SDS–PAGE gel and digested with trypticases. Digested peptides were performed on a Thermo Q-Exactive high resolution mass spectrometer (Thermo Scientific, Waltham, MA, USA). Source parameters were 2 kV spray voltage and 320 °C capillary temperature. Data obtained from the mass spectrometer were preprocessed with Mascot Distiller 2.4 for peak picking. The resulted peak lists were searched against Swissprot database using Mascot 2.4 search engine.

## Additional information

**Accession codes**: RNA-seq data associated with this study have been deposited in the NCBI sequence read archive under accession codes SRP062359.

**How to cite this article:** Kong, L. *et al.* Degradation of the ABA co-receptor ABI1 by PUB12/13 U-box E3 ligases. *Nat. Commun.* 6:8630 doi: 10.1038/ncomms9630 (2015).

## Supplementary Material

Supplementary Information Supplementary Figures 1-12, Supplementary Tables 1-3

Supplementary Data 1 LC-MS/MS data of ABI1-Flag co-purified proteins

Supplementary Data 2 The genes induced by ABA treatment for 1 h in the wild type (Col), and their relative expression levels in pub12 pub13 and abi1-1 (Col)

Supplementary Data 3 The genes induced by ABA treatment for 3 h in the wild type (Col), and their relative expression levels in pub12 pub13 and abi1-1 (Col)
