# Supplementary material for: Degradation of the ABA co-receptor ABI1 by PUB12/13 U-box E3 ligases
Source: Nat Commun. 2015 Oct 20;6:8630. doi: 10.1038/ncomms9630 (PMC4667695; doi:10.1038/ncomms9630)
Supplement: Supplementary Information — Supplementary Figures 1-12, Supplementary Tables 1-3 [file ncomms9630-s1.pdf]

Supplementary Figures 1-12

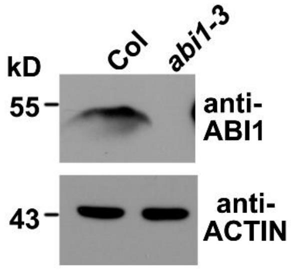

Supplementary Figure 1. The specificity of anti-ABI1 antibody. Total Proteins extracted from the wild type seedlings or *abi1-3* null mutant seedlings were used for immunoblotting analysis with anti-ABI1 antibody. ACTIN was used as a loading control.

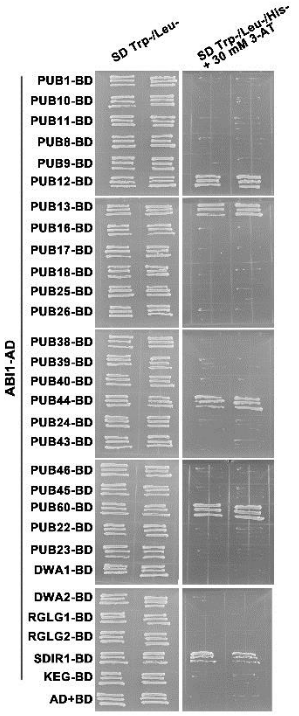

Supplementary Figure 2. Yeast two-hybrid assay to detect ABI1 interaction with selected proteins. AD: Gal4-activation domain; BD: Gal4 DNA-binding domain. 2D: synthetic dropout medium without Trp and Leu; 3D: interaction medium without Trp, Leu and His.

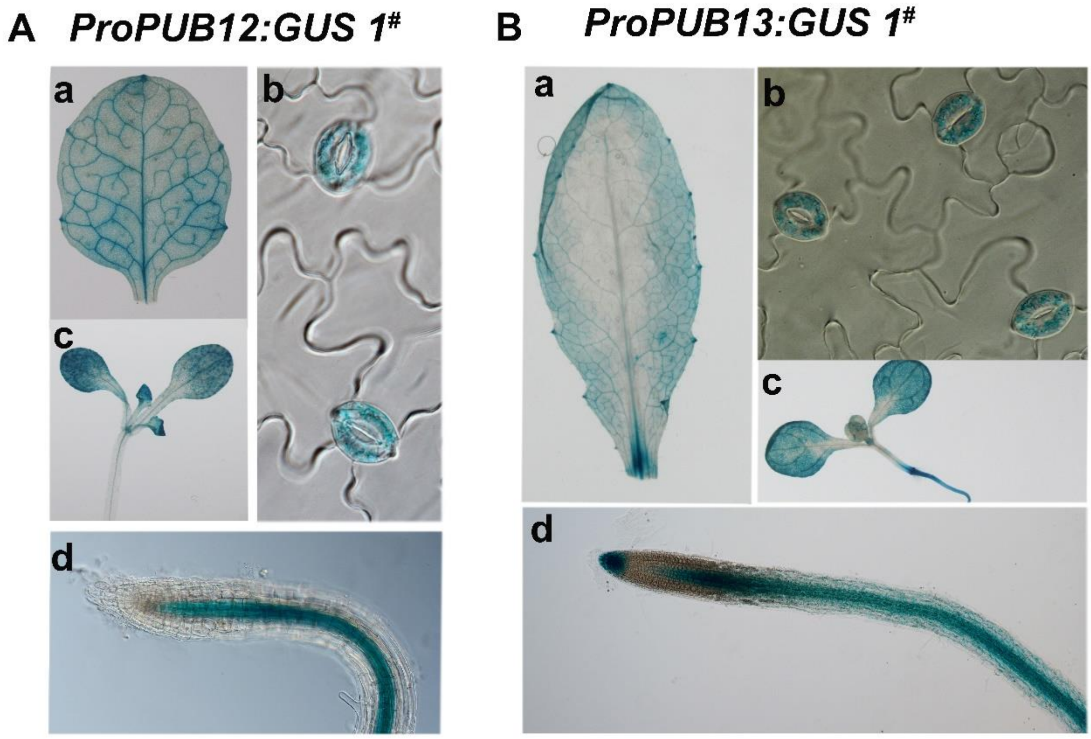

Supplementary Figure 3. The expression patterns of *ProPUB12:GUS* and *ProPUB13:GUS* in transgenic plants.

A. *ProPUB12:GUS* expression in a transgenic line 1. a, a true leaf; b, guard cells; c, a young seedling; d, a primary root.

B. *ProPUB1b:GUS* expression in a transgenic line 1. a, a true leaf; b, guard cells; c, a young seedling; d, a primary root.

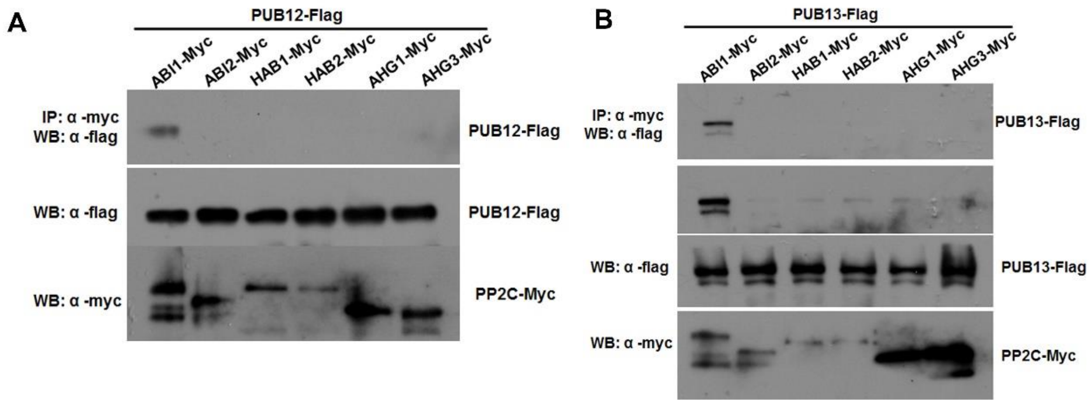

Supplementary Figure 4. PUB12/PUB13 do not interact with ABI2, HAB1, HAB2, AHG1, or AHG2 in a Co-IP assay.

Protoplasts were co-expressed with different PP2Cs (Myc tag) and PUB12- (A) or PUB13-Flag (B). Co-IP was carried out with anti-Myc agarose, and Immunoblotting analysis was done with anti-Flag and anti-Myc antibody.

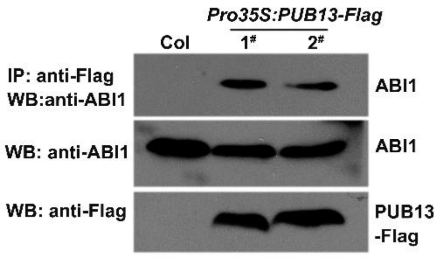

Supplementary Figure 5. ABI1 co-immunoprecipitates PUB13-Flag protein. Co-IP was carried out with anti-Flag agarose from total proteins of *Pro35S:PUB13-Flag* transgenic seedlings, and immunoblotting analysis was done with anti-Flag and anti-ABI1 antibody.

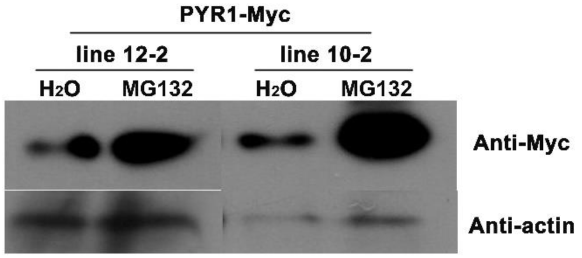

Supplementary Figure 6. PYR1 degradation by the 26S proteasome pathway.

Treatment with the 26S proteasome inhibitor MG132 greatly increases the level of PYR1-Myc. Two transgenic lines overexpressing PYR1-Myc were treated with 50 μM MG132 or H_2O for 6 h. Equal protein loading was demonstrated by Immunoblotting analysis with anti-ACTIN antibody.

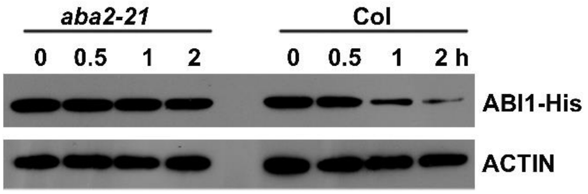

Supplementary Figure 7. ABI1 protein is more stable in *aba2-21* than the wild type in the cell-free 26S proteasome assay. Total proteins were extracted from ABA deficient mutant *aba2-21* (contains less than 10% ABA of the wild type) and the wild type. ABI1-His protein purified from *E. coli* was added together with the extracted proteins into the ATP containing cell-free solution, and incubated for 0, 0.5, 1 and 2 h. Then the proteins were used for immunoblotting analysis using anti- His antibody. ACTIN was used as a loading control.

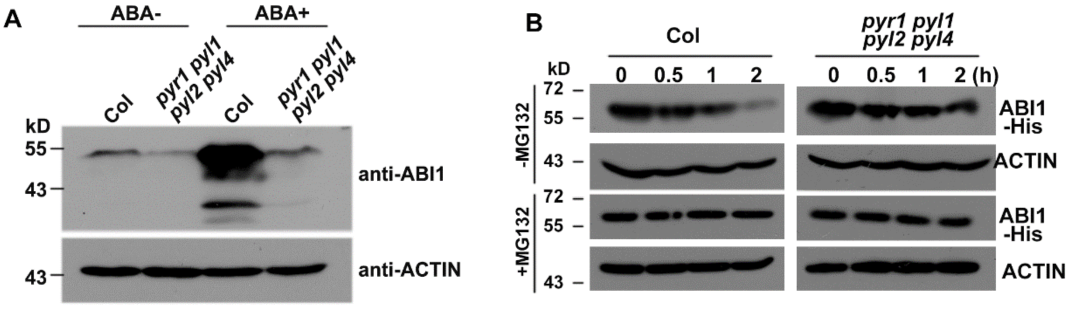

Supplementary Figure 8. ABI1 protein is lower in *pyr1 pyl1 pyl2 pyl4* quadruple mutant than in the wild type, but more stabilized in protein solution from *pyr1 pyl1 pyl2 pyl4* quadruple mutant than from the wild type.

A. ABI1 protein is accumulated less in *pyr1 pyl1 pyl2 pyl4* quadruple mutant than in the wild type in both no ABA treatment and ABA treatment. Total proteins extracted from 7-day-old seedlings treated with 50 M ABA for 6 hr or without ABA were used for immunoblotting analysis with anti-ABI1 antibody. ACTIN was used as a loading control.

B. Comparison of ABI1-His stability between *pyr1 pyl1 pyl2 pyl4* quadruple mutant and the wild type in a cell free assay. ABI1-His protein purified from *E. coli* was added to the ATP containing cell-free solution with total proteins from *pyr1 pyl1 pyl2 pyl4* quadruple mutant or the wild type, respectively, in absence (upper) or presence (lower) of MG132 (50 μM). The proteins were used for immunoblotting analysis after cultured for different times. ACTIN was used as a loading control.

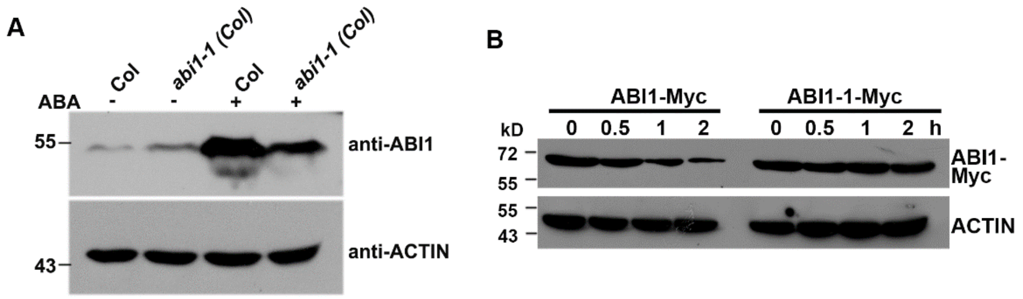

Supplementary Figure 9. ABI1-1 protein level in *abi1-1* mutant and ABI1-1 stability in a cell free assay.

A. ABI1 protein is higher in *abi1-1* (Col) than in the wild type without ABA treatment, but lower with ABA treatment. Proteins extracted from seedlings treated with 50 µM ABA for 6 hr or without ABA treatment were used for immunoblotting analysis with anti-ABI1 antibody. ACTIN was used as a loading control.

B. The degradation of the mutated ABI1^{G180D} (ABI1-1) is greatly reduced in the cell-free 26S proteasome assay. The protoplasts were transiently expressed with the *Pro35S:ABI1-Myc* or *Pro35S:ABI1-1-Myc* plasmid in the wild type (Col). The total extracted proteins were equally divided into four parts, which were incubated in in 26S proteasome degradation buffer for different times. Immunoblotting analysis was carried out with anti-Myc antibody. ACTIN was used as a loading control.

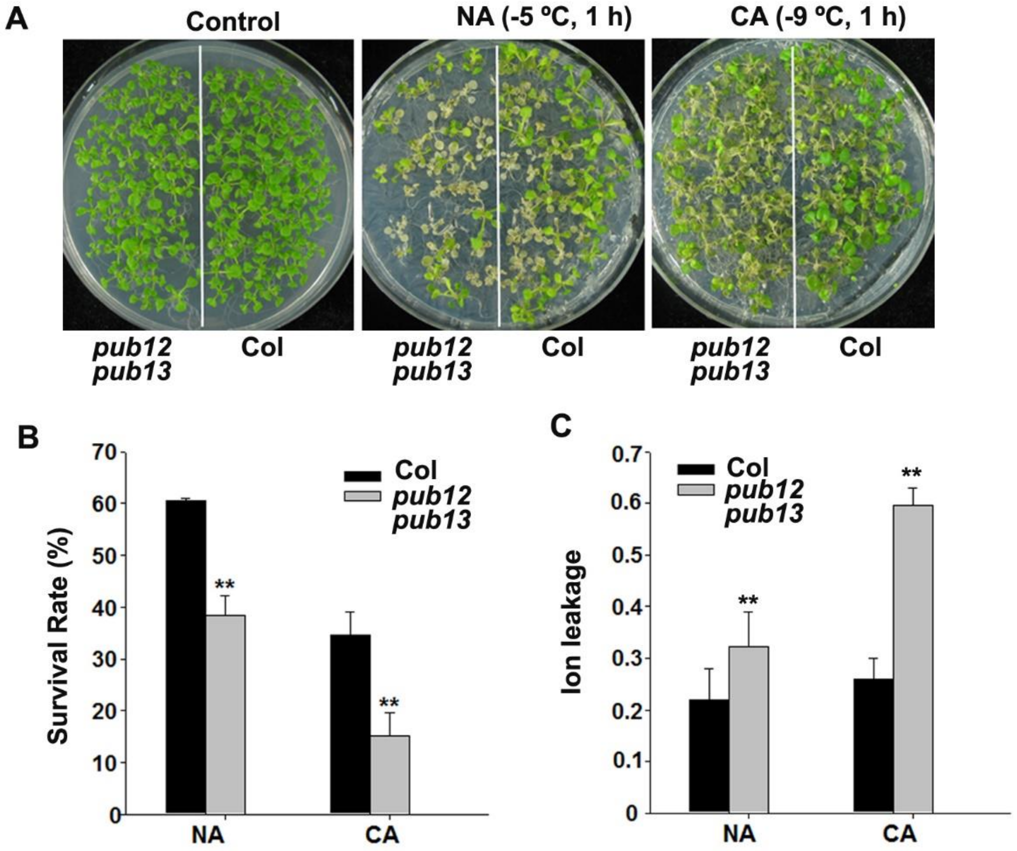

Supplementary Figure 10. ***pub12 pub13*** **mutants are more sensitivity to freezing stress than wild type (Col).**

Comparison of freezing tolerance of *pub12 pub13* mutant and the wild type (Col). Two-week seedlings grown on MS plates at 22 ℃ were treated at -5 ℃ for 1 h (non-acclimation, NA), or first treated at 4 ℃ for 4 days, and then treated at -9 ℃ for 1 h (Cold-acclimation, CA). After 3 days at 22 ℃, the images were taken (A), and survival rates (B) and ion leakage (C) were measured. Data are means of three replicates ± SD, and the asterisks indicate significant differences compared with the Col under the same treatment conditions (**$p < 0.01$, t test).

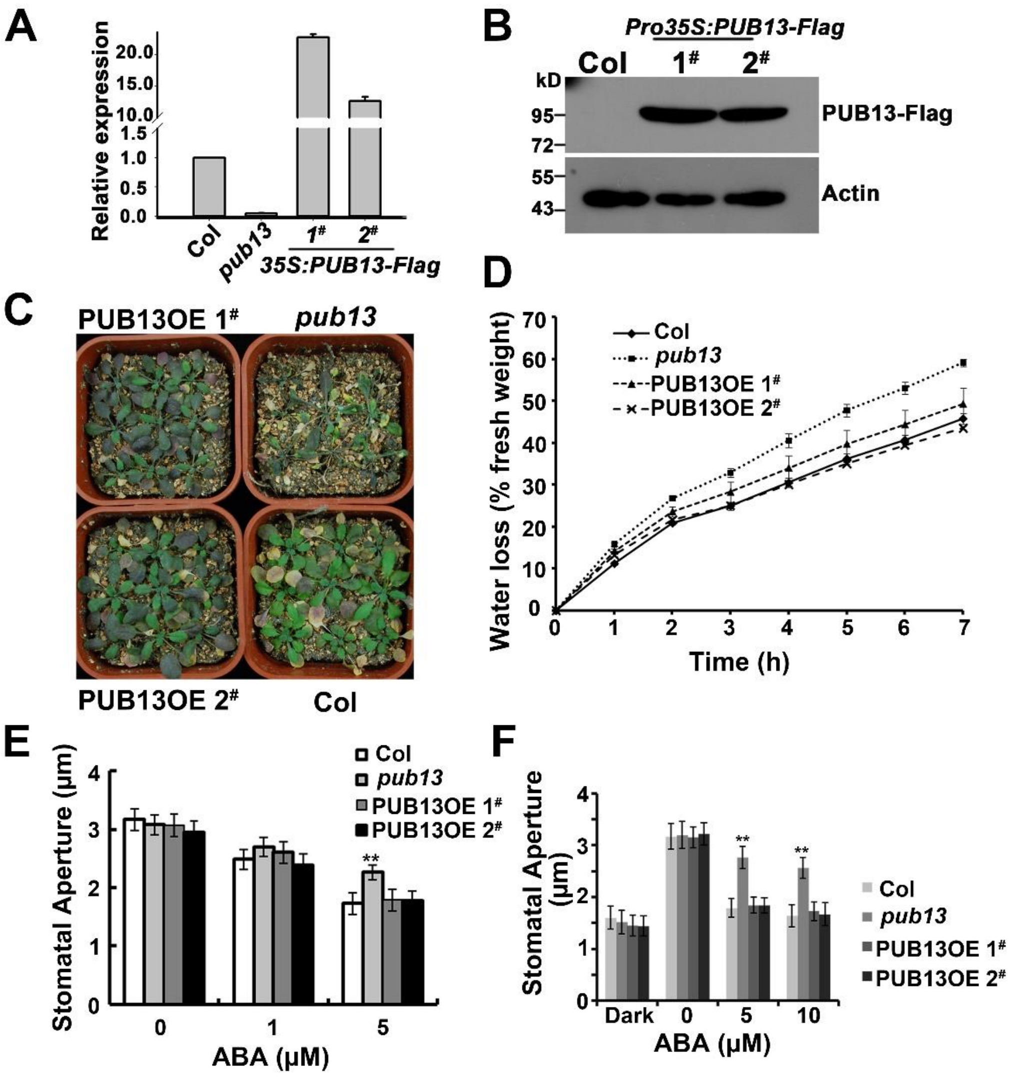

Supplementary Figure 11. The phenotypic analyses of PUB13 overexpression plants in ABA responses.

A. The relative expression of *PUB13* by QRT-PCR in two independent transgenic Arabidopsis lines.

B. Immunoblotting analysis of PUB13 protein in two independent transgenic *Arabidopsis* lines.

C. Phenotypic analysis of drought stress among PUB13 overexpression lines, *pub13* mutant and the wild type when growing in soil. Water was withheld from 19-day-old

seedlings growing in soil; after 10 days without water, the seedlings were photographed.

D. Water loss analysis of detached leaves from PUB13 overexpression lines, *pub13* mutant and the wild type. Three independent experiments were done with similar results. Values are means ± SE of three replicates (40 leaves from one pot were measured per replicate) from one representative experiment.

E. Comparison of ABA-induced stomatal closure among PUB13 overexpression lines, *pub13* mutant and the wild type. Leaf epidermal peels were treated with MES buffer for 2 h under strong light to fully open stomata. After the peels were incubated with different concentrations of ABA for 2 h, stomatal apertures were measured with Image J. Values are means ± SE of three replicates (120-150 stomata from one seedling in each replicate) from one representative experiment; three independent experiments were done with similar results (**: $P < 0.01$, *: $P < 0.05$).

F. Comparison of ABA-inhibited stomatal opening among PUB13 overexpression lines, *pub13* mutant and the wild type. Four-week-old seedlings were kept in darkness for 24 h. Different concentrations of ABA were then added, and seedlings were kept under strong light for 3 h before stomatal apertures were measured. Values are means ± SE of three replicates (120-150 stomata from one seedling in each replicate) from one representative experiment; three independent experiments were done with similar results (**: $P < 0.01$, *: $P < 0.05$).

Supplementary Figure 12. The original western blot images and independent experiment repeats used in text. All experiments were independently repeated at least one time. For all quantitative data, the experiments were independently repeated at least two times (total three times).

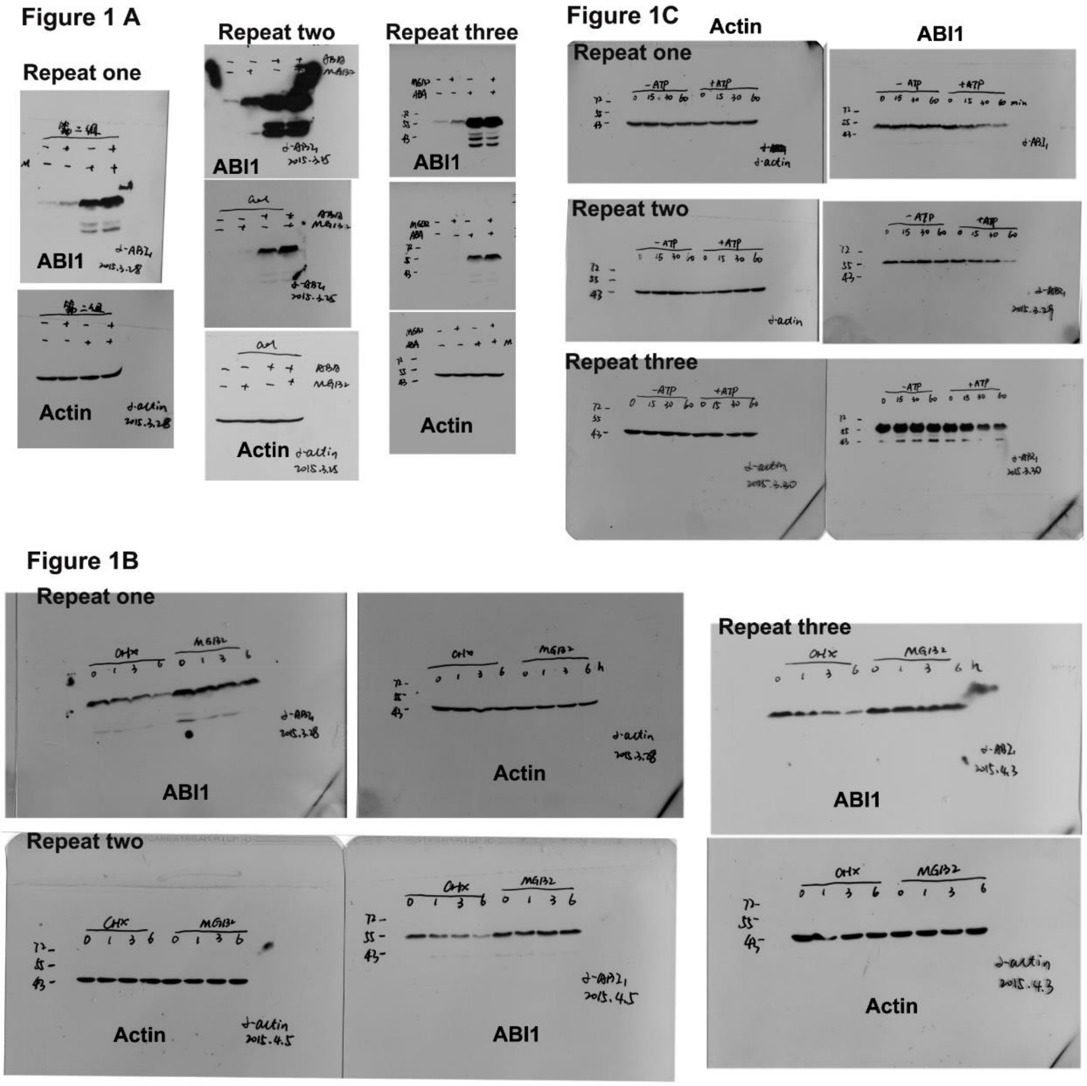

Figure 1. Original images and independent experiment repeats.

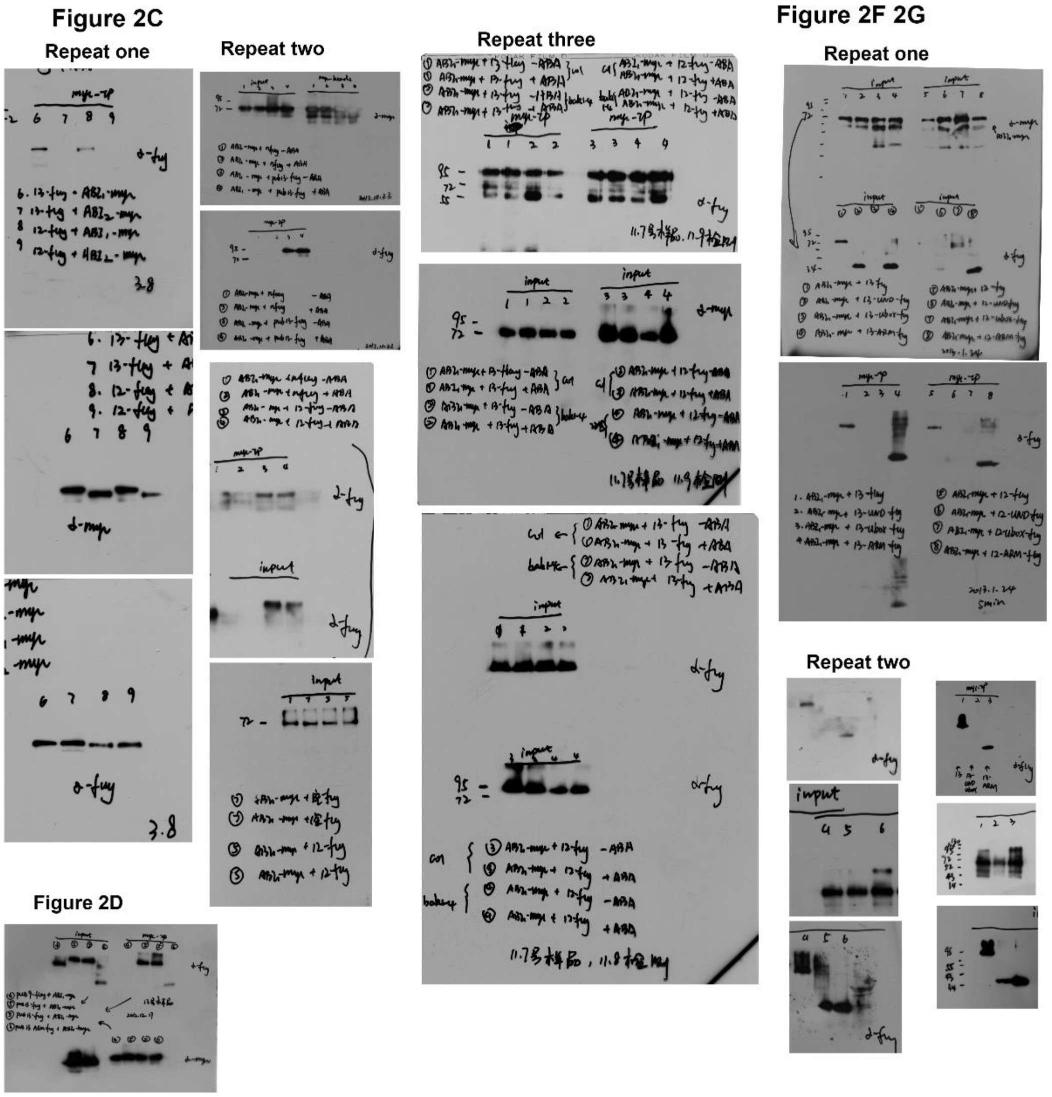

Figure 2. Original images and independent experiment repeats.

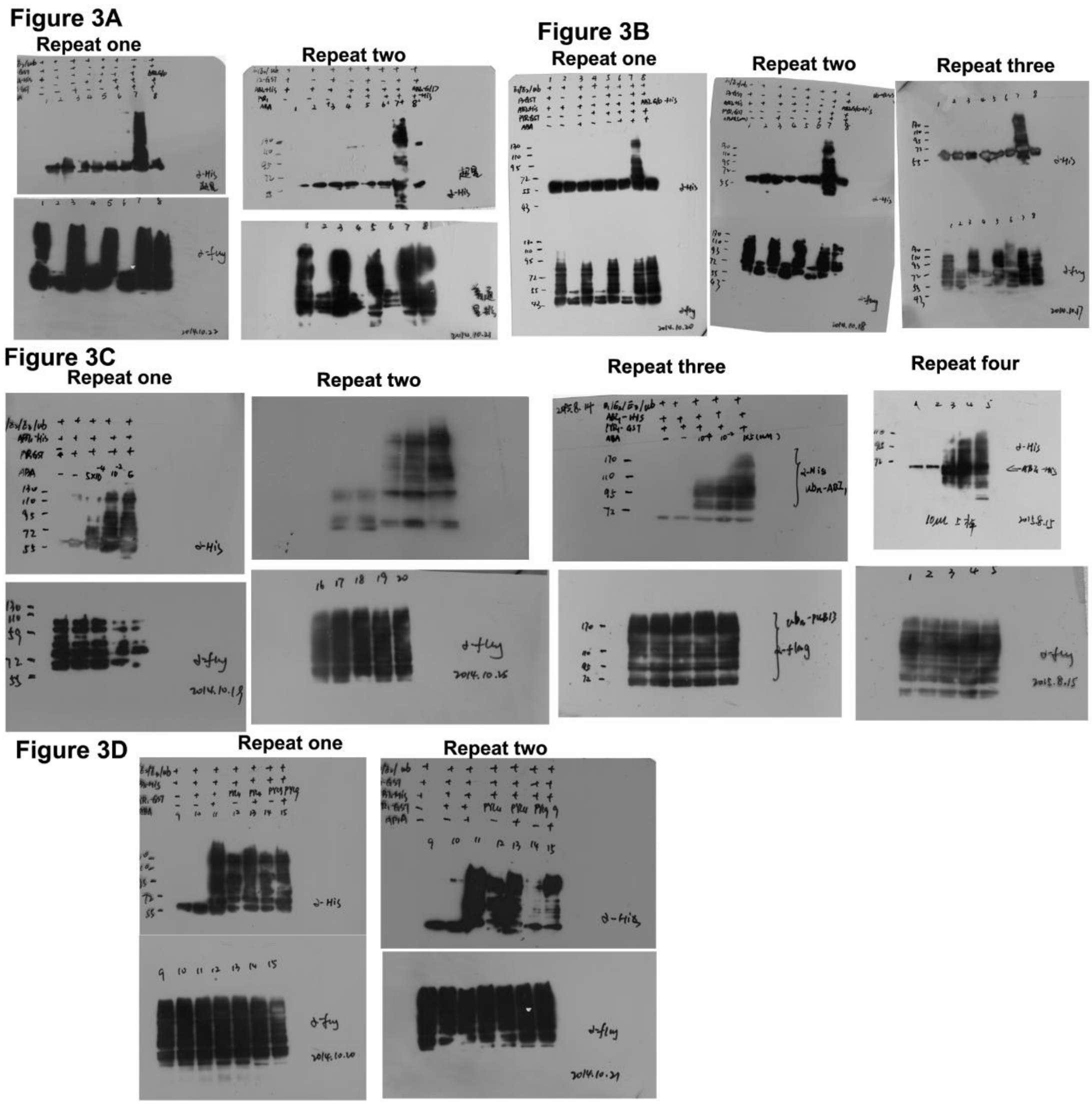

Figure 3. Original images and independent experiment repeats.

Figure 4a

Repeat one Repeat two Repeat three

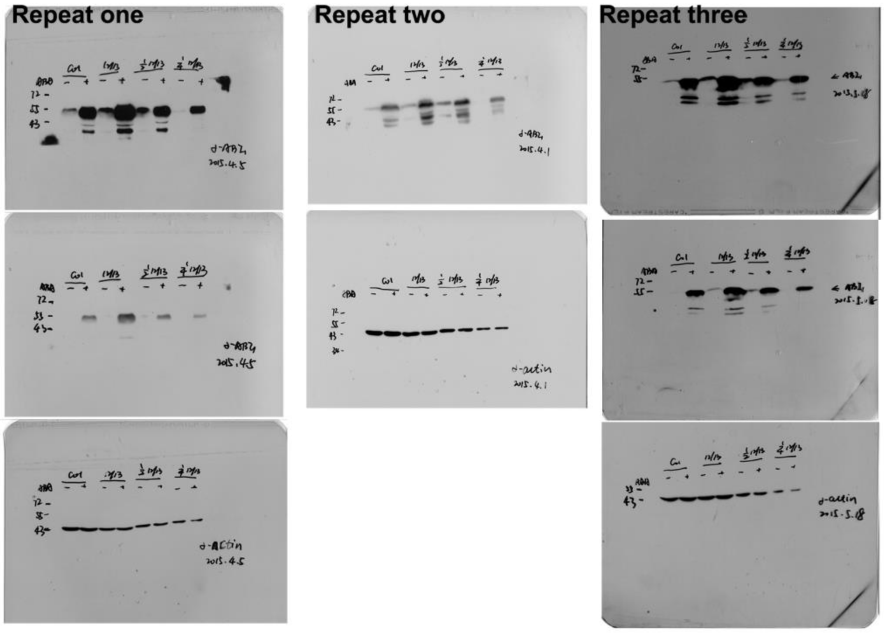

Figure 4 c

Repeat one Repeat two Repeat three

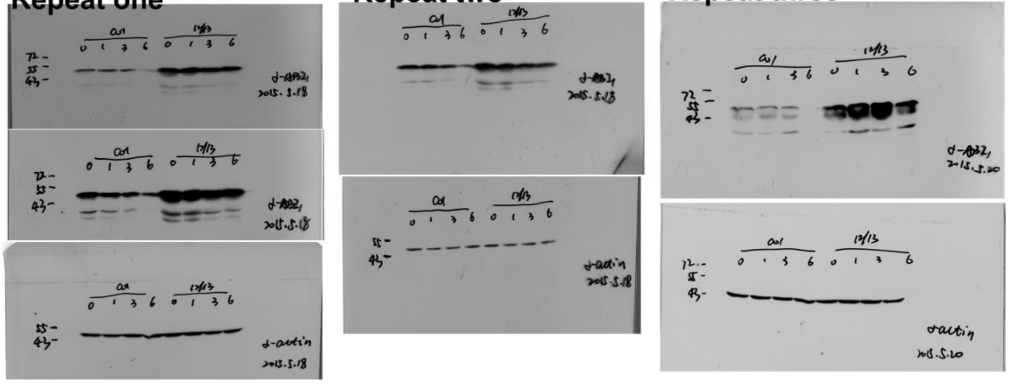

Figure 4 e

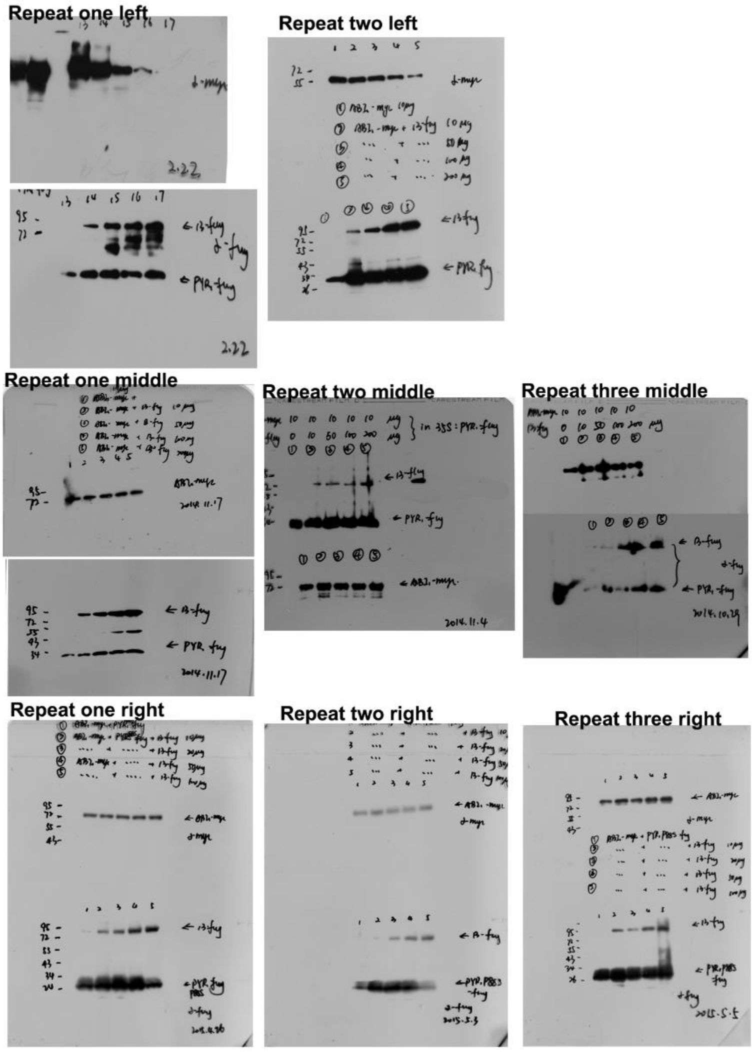

Figure 4. Original images and independent experiment repeats.

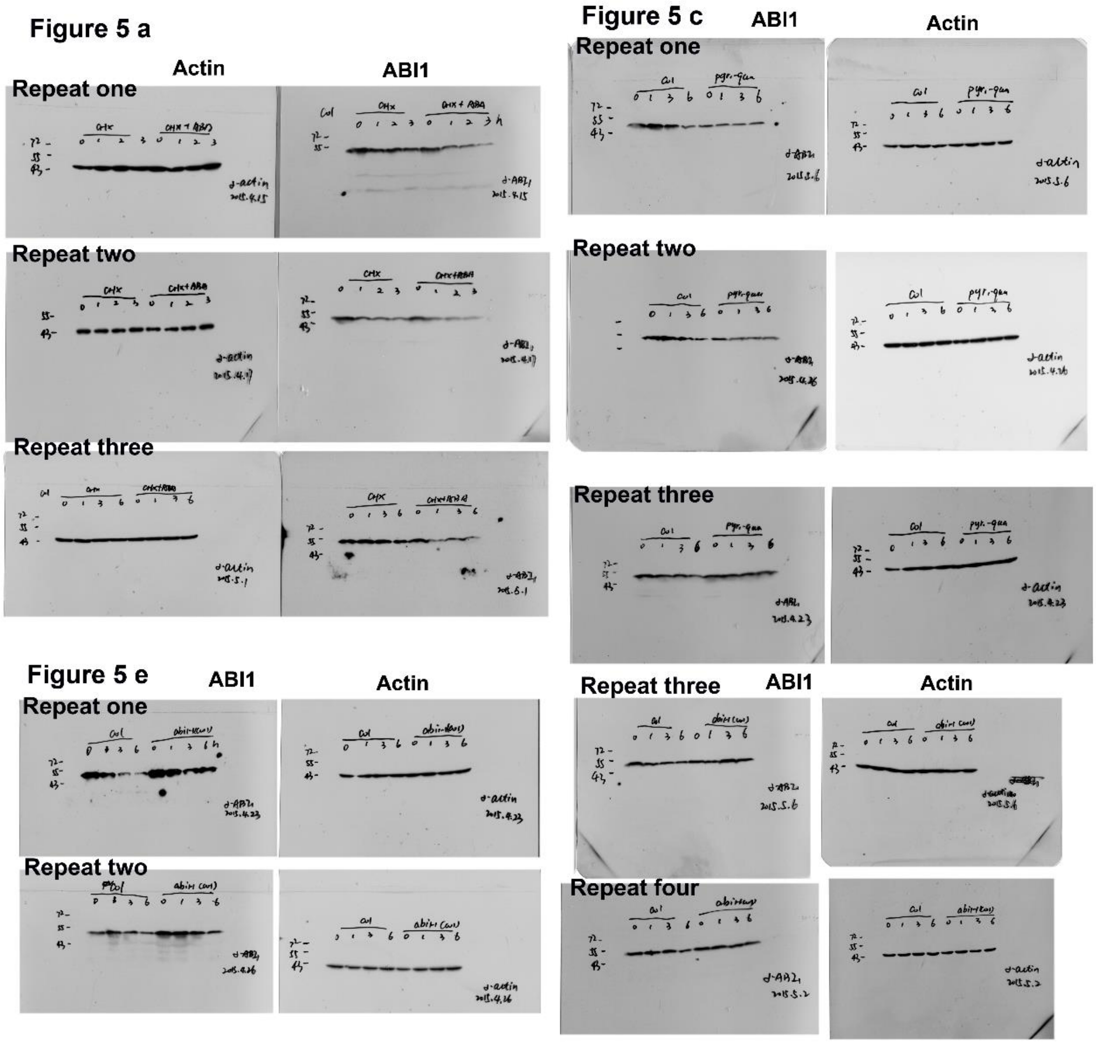

Figure 5. Original images and independent experiment repeats.

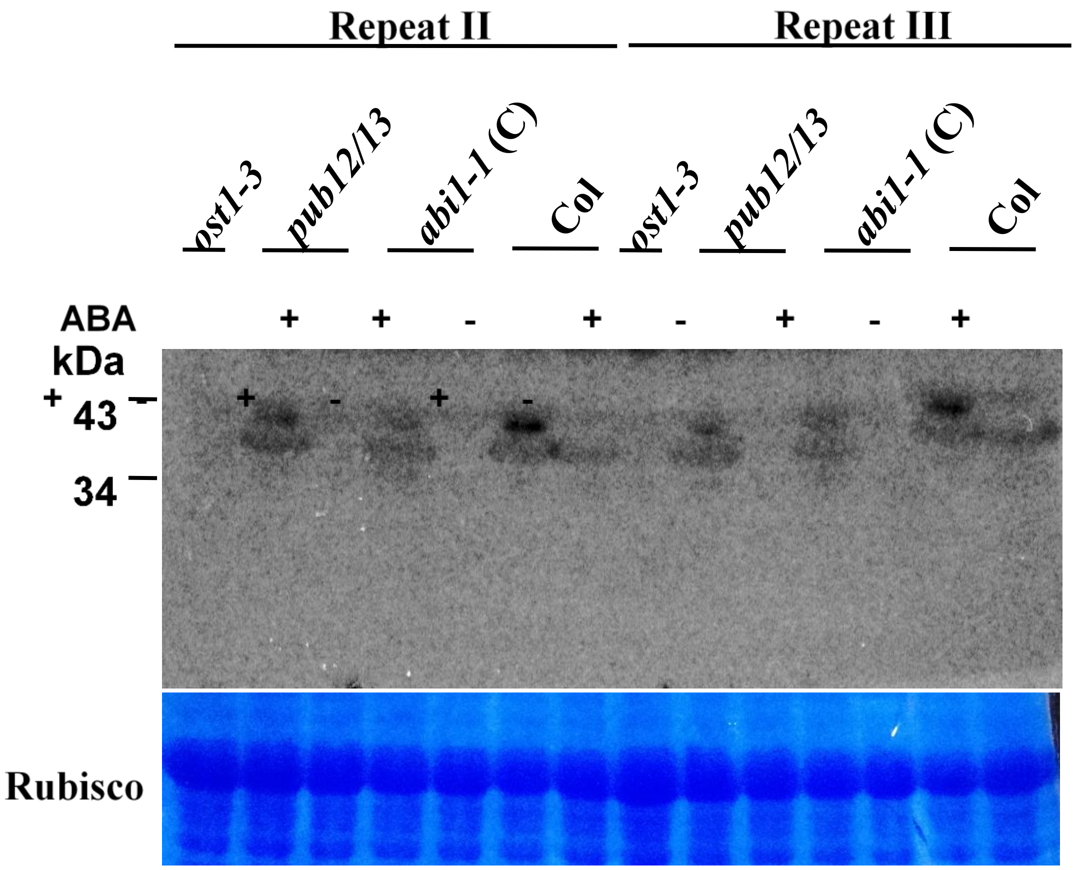

Figure 6a. The image in Figure 6a is the original one. Here are two other independent Experiments. Seedlings from different MS plates were independently treated with ABA or without ABA at different day time, and proteins were separately isolated, but run on the same gel.

Supplementary Figure 1

Repeat one

Repeat two

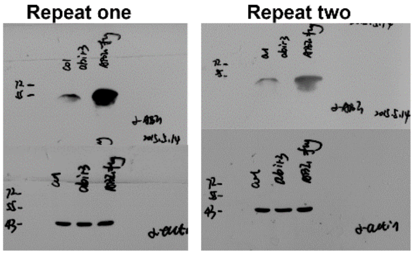

Supplementary Figure 4

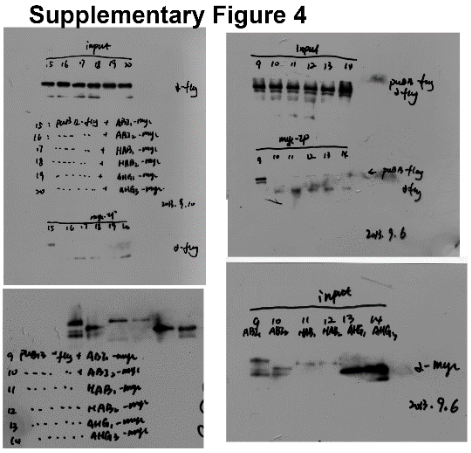

Supplementary Figure 5

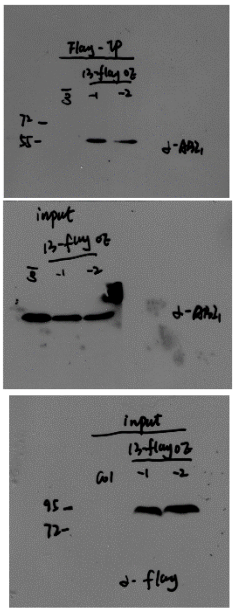

Supplementary Figure 7

Repeat one

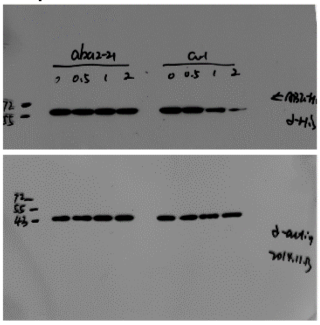

Repeat two

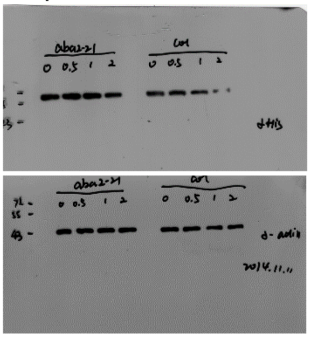

Supplementary Figure 1, 4, 5, 7. Original images and independent experiment repeats.

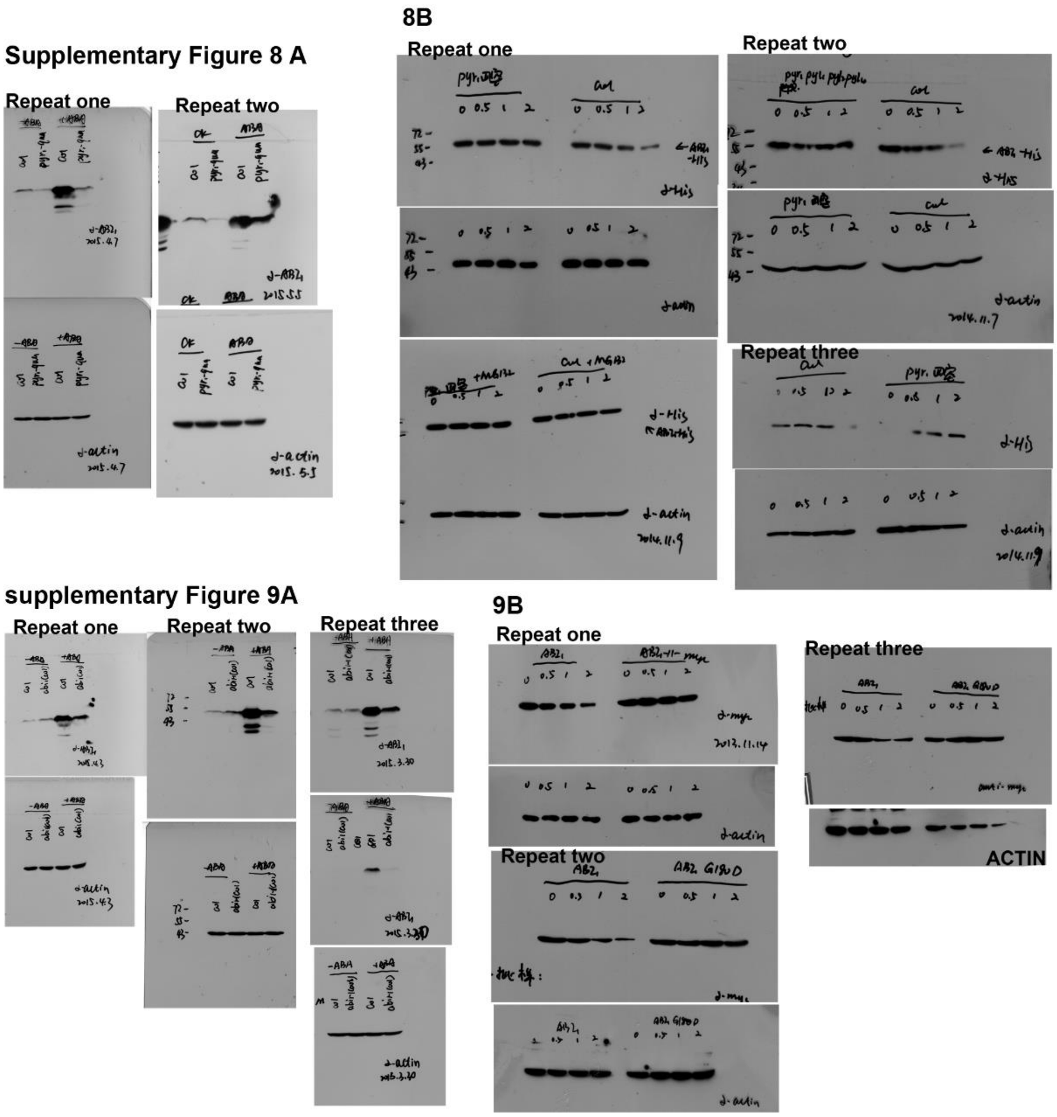

Supplementary Figure 8, 9. Original images and independent experiment repeats.

Supplementary Table 1. Primers used in this study

Primers for confirming T-DNA insertions

Primer name	sequence
pub12-LP	5'-TAACCACAGCTACCCAAAACG-3'
pub12-RP	5'-TAATTTCCTAATTTGGCCGTG-3'
pub13-LP	5'-AAGAGGTATGGCTCCAGCTTC-3'
pub13-RP	5'-ACGTGCTTTGTTTTGCTATGG-3

Primers for RT-PCR analysis

Primer name	sequence
RD29A –RT-F	5'-GACGAGTCAGGAGCTGAGCTG-3'
RD29A –RT-R	5'-CGATGCTGCCTTCTCGGTAGAG-3'
RD29B –RT-F	5'-TGTCTTCTGACCACACCAAACCCATTG-3'
RD29B –RT-R	5'-CCACCAGGAGCAAACGTCCTAGTCA-3'
PUB12-RT-F	5'- TGTTAAGCAATCGCTTCTTCATGCT -3'
PUB12-RT-R	5'- TACAACTCATCATCATACACATCGCCA -3'
PUB13-RT-F	5'- TCGCTGGAAATGATGAGAGATCCA -3'
PUB13-RT-R	5'- CGTACGCAAGTCTCCACATAAGATCT -3'
Actin4-F	5'- AGCACTTGCACCAAGCAGCATG -3'
Actin4-R	5'- ACGATTCCTGGACCTGCCTCATC -3'

Primers for ubiquitination and Co-IP assay

Primer name	sequence
PUB12-GST-*Bam*H I F	5'- ACGGGATCCATGGCGAAATCAGAGAAACACAAATTAGCTCA -3'
PUB12-GST-*Xho* I R	5'-CGCTCGAGTTTAGAAATGATGAATTTGGTTTTTGCAGGCA -3'
PUB13-GST-*Eco*R I F	5'- ACGGAATTCATGGAGGAAGAGAAAGCTTCTGCT -3'
PUB13-GST-*Xho* I R	5'- CGCTCGAGAGTATCTGCAGCTTCTGTGGTGGAT -3'
UBC8-GST-*Eco*R I F	5'- AGCGAATTCATGGCTTCGAAACGGATCTTGAAGGA-3'
UBC8-GST-*Not* I R	5'- ACGGCGGCCGCTTAGCCCATGGCATACTTCTGAGTCCA -3'
ABI1-His- *Bam*H I F	5'-CGCGGATCCATGGAGGAAGTATCTCCGGCGATC -3'
ABI1-His- *Sal* I R	5'-CGCGTCGACGTTCAAGGGTTTGCTCTTGAGTTTCCTC -3'
PYR1-GST-*Bam*H I F	5'- GCGGATCCATGCCTTCGGAGTTAACACCAGAAGAAC -3'
PYR1-GST- *Sal* I R	5'- GCGTCGACTCACGTCACCTGAGAACCACTTCC -3'
PYL4-GST-*Bam*H I F	5'- GCGGATCCATGCTTGCCGTTCACCGTCC -3'
PYL4-GST- *Sal* I R	5'- GCGTCGACTCACAGAGACATCTTCTTCTTGCTCTCAG -3'
PYL9-GST-*Bam*H I F	5'- GCGGATCCATGATGGACGGCGTTGAAGGC -3'

PYL9-GST- *Sal* I R	5'- GCGTCGACTCACTGAGTAATGTCCTGAGAAGCCAATC -3'
ABI1-Myc- *Sal* I F	5'- CGCGTCGACATGGAGGAAGTATCTCCGGCGATC -3'
ABI1-Myc-*Kpn* I R	5'-CGCGGTACCGCAGTTCAAGGGTTTGCTCTTGAGTTTCC -3'
PYR1-Myc-*Sal* I F	5'- CGCGTCGACATGCCTTCGGAGTTAACACCAGAAGAAC -3'
PYR1-Myc-*Kpn* I R	5'- CGCGGTACCCGTCACCTGAGAACCACTTCCGT -3'
PUB13-Flag-*Sal* I F	5'- ACGGTCGACATGGAGGAAGAGAAAGCTTCTGCT -3'
PUB13-Flag-*Kpn*I R	5'- CGGGTACCAGTATCTGCAGCTTCTGTGGTGGAT -3'
PUB12-Flag-*Sal* I F	5'-AGCGTCGACATGGCGAAATCAGAGAAACACAAATTA -3'
PUB12-Flag-*Kpn* I R	5'- CGCGGTACCGATTAGGGAGATTTGATCTTCC-3'
PUB12-UND/U-box-Flag-*Spe* I F	5'- ACGACTAGTATGGCGAAATCAGAGAAACACAAATTAGCT -3'
PUB12-UND/U-box-Flag- *Sal* I R	5'- CGGTCGACTTCGATGCCATTGGACTCACACC -3'
PUB12-ARM- Flag-*Spe*I F	5'- ACG ACTAGTAACAATCATAACCGAGTCGCCATT -3'
PUB12-ARM- Flag - *Sal* I R	5'- CGGTCGACGATTAGGGAGATTTGATCTTCC -3'
PUB13-UND/U-box-Flag-*Spe* I F	5'- ACGACTAGTATGGAGGAAGAGAAAGCTTCTGCT -3'
PUB13-UND/U-box-Flag- *Sal* I R	5'- CG GTCGACCTCAATATCGTTGGCCTCGCACC -3
PUB13-ARM- Flag-*Spe* I F	5'- ACGACTAGTTATGTTCTCCGTAGTCTCATAGCTCAGT -3'
PUB13-ARM- Flag - *Sal* I R	5'- CGCCCGGGAGTATCTGCAGCTTCTGTGGTGGAT -3'
PUB9-Flag-*Xba* I F	5'- CGCTCTAGAATGGCGAAGACCGGTGTTTTCGAT -3'
PUB9-Flag-*Xma* I R	5'-ACGCCCGGGAGGCTGTATGAGTGAGATTCATAGCTTTTCTCAGT -3'

Primers for Yeast two-hybrid assay analysis

Primer name	sequence
PUB12-BD-*Eco*R I F	5'- ACGGAATTCATGGCGAAATCAGAGAAACACAAATTAGCTCA -3'
PUB12-BD-*Sal* I R	5'- CGGTCGACTTTAGAAATGATGAATTTGGTTTTTGCAGGCAACT -3'
PUB13-BD-*Eco*R I F	5'- ACGGAATTCATGGAGGAAGAGAAAGCTTCTGCT -3'
PUB13-BD-*Pst* I R	5'- CGCTGCAGAGTATCTGCAGCTTCTGTGGTGGT -3'

Primers for LUC assay analysis

Primer name	sequence
PUB12-LUC-*Kpn* I F	5'- CGCGGTACCATGGCGAAATCAGAGAAACACAAATTAGCTCA -3'
PUB12-LUC-*Sal* I R	5'- CGCGTCGACGATTAGGGAGATTTGATCTTCC-3'
PUB12-ARM-LUC-*Kpn* I F	5'- CGCGGTACCTATGTTCTAAGAAGCCTTATAGCTCAATGGT -3'
PUB12-ARM-LUC-*Sal* I R	as PUB12-LUC *Sal* I R
PUB13-LUC-*Kpn* I F	5'- ACGGGTACCATGGAGGAAGAGAAAGCTTCTGCT -3'
PUB13-LUC-*Sal* I R	5'- CGGTCGACAGTATCTGCAGCTTCTGTGGTGGAT -3'
PUB13-ARM-LUC-*Kpn* I F	5'- ACGGGTACCTATGTTCTCCGTAGTCTCATAGCTCAGT -3'
PUB13-ARM-LUC-*Sal* I R	as PUB13-LUC *Sal* I R

ABI1-LUC-*Kpn* I F	5'-CGCGGTACCATGGAGGAAGTATCTCCGGCGATC -3'
ABI1-LUC-*Sal* I R	5'-CGCGTCGACGCAGTTCAAGGGTTTGCTCTTGAGTTTCC -3'

Primers for promoters:

Primer name	sequence
PUB12-Pro *Pst* I F	5'- GCGCTGCAGAGAAATGCGGTGCAGTTCAAATAGT -3'
PUB12-Pro *Eco*R I R	5'- CGCGAATTCTTTGTGTTTCTCTGATTTCGCCAGT -3'
PUB13-Pro *Pst* I F	5'- GCGCTGCAGTCAATCTCTAGCTGATTACATCAACATAAGA -3'
PUB13-Pro *Eco*RI R	5'-CGCGAATTCTTCCTCCATTGAATTGATTCTTCTCTGT -3'
ABI1-pro *Bam*HI F	5'-CGCGGATCCAATTTCTACTGGCCAAGTGAAGGCC -3'
ABI1-pro *Pst*I R	5'-GCCTGCAGTAACGGTAAAGATTTGATCTTTTTCTCTGGTTGTGA -3'

Supplementary Table 2. Number of reads per sample and alignment statistics

Study	Project	Sample	Experiment	RunID （now）	Data file	Data file
SRP062359	PRJNA292748	SRS1030197	SRX1144162	SRR2162785	gonglab_11_1.clean.fq.gz (Col-ABA 0 h repeat 1-1)	gonglab_11_2.clean.fq.gz (Col-ABA 0 h repeat 1-2)
SRP062359	PRJNA292748	SRS1030197	SRX1144162	SRR2170688	gonglab_12_1.clean.fq.gz (Col-ABA 0 h repeat 2-1)	gonglab_12_2.clean.fq.gz (Col-ABA 0 h repeat 2-2)
SRP062359	PRJNA292748	SRS1030197	SRX1144164	SRR2163021	gonglab_7_1.clean.fq.gz (Col-ABA 1 h repeat 1-1)	gonglab_7_2.clean.fq.gz (Col-ABA 1 h repeat 1-2)
SRP062359	PRJNA292748	SRS1030197	SRX1144164	SRR2170691	gonglab_8_1.clean.fq.gz (Col-ABA 1 h repeat 2-1)	gonglab_8_2.clean.fq.gz (Col-ABA 1 h repeat 2-2)
SRP062359	PRJNA292748	SRS1030197	SRX1144165	SRR2163037	gonglab_9_1.clean.fq.gz (Col-ABA 3 h repeat 1-1)	gonglab_9_2.clean.fq.gz (Col-ABA 3 h repeat 1-2)
SRP062359	PRJNA292748	SRS1030197	SRX1144165	SRR2170764	gonglab_10_1.clean.fq.gz (Col-ABA 3 h repeat 2-1)	gonglab_10_2.clean.fq.gz (Col-ABA 3 h repeat 2-2)
SRP062359	PRJNA292748	SRS1041128	SRX1144166	SRR2163041	gonglab_17_1.clean.fq.gz (*abi1*(Col)-ABA 0 h repeat 1-1)	gonglab_17_2.clean.fq.gz (*abi1*(Col)-ABA 0 h repeat 1-2)
SRP062359	PRJNA292748	SRS1041128	SRX1144166	SRR2170765	gonglab_18_1.clean.fq.gz (*abi1*(Col)-ABA 0 h repeat 2-1)	gonglab_18_2.clean.fq.gz (*abi1*(Col)-ABA 0 h repeat 2-2)
SRP062359	PRJNA292748	SRS1041128	SRX1144167	SRR2163045	gonglab_13_1.clean.fq.gz (*abi1*(Col)-ABA 1 h repeat 1-1)	gonglab_13_2.clean.fq.gz (*abi1*(Col)-ABA 1 h repeat 1-2)
SRP062359	PRJNA292748	SRS1041128	SRX1144167	SRR2170766	gonglab_14_1.clean.fq.gz (*abi1*(Col)-ABA 1 h repeat 2-1)	gonglab_14_2.clean.fq.gz (*abi1*(Col)-ABA 1 h repeat 2-2)
SRP062359	PRJNA292748	SRS1041128	SRX1144168	SRR2163046	gonglab_15_1.clean.fq.gz (*abi1*(Col)-ABA 3 h repeat 1-1)	gonglab_15_2.clean.fq.gz (*abi1*(Col)-ABA 3 h repeat 1-2)
SRP062359	PRJNA292748	SRS1041128	SRX1144168	SRR2170767	gonglab_16_1.clean.fq.gz (*abi1*(Col)-ABA 3 h repeat 2-1)	gonglab_16_2.clean.fq.gz (*abi1*(Col)-ABA 3 h repeat 2-2)

Supplementary Table 3. The antibodies used in this study

antibody or beads name	manufacturer	Cat. NO.	diluted	monoclonal or polyclonal antibody
Anti-c-Myc antibody	Sigma-Aldrich	M4439	1/5000	monoclonal antibody
Anti-Flag antibody	Sigma-Aldrich	F3165	1/5000	monoclonal antibody
Anti-c-Myc Agarose Affinity Gel antibody	Sigma-Aldrich	A7470	none	monoclonal antibody
Anti-Flag Affinity Gel	Sigma-Aldrich	A2220	none	monoclonal antibody
Anti-His antibody	Abmart	#M20001	1/5000	monoclonal antibody
Anti-Actin antibody	Abmart	#M20009	1/2000	monoclonal antibody
Anti-ABI1 antibody	self-made	self-made	1/2000	polyclonal antibody
Goat Anti-Mouse IgG-HRP	Abmart	#M21001	1/10000	none
Goat Anti-Rabbit IgG-HRP	Abmart	#M21002	1/10000	none
